# LncRNA RUNX1-IT1 which is downregulated by hypoxia-driven histone deacetylase 3 represses proliferation and cancer stem-like properties in hepatocellular carcinoma cells

**DOI:** 10.1038/s41419-020-2274-x

**Published:** 2020-02-05

**Authors:** Liankang Sun, Liang Wang, Tianxiang Chen, Yu Shi, Bowen Yao, Zhikui Liu, Yufeng Wang, Qing Li, Runkun Liu, Yongshen Niu, Kangsheng Tu, Qingguang Liu

**Affiliations:** 1 Department of Hepatobiliary Surgery, The First Affiliated Hospital of Xi’an Jiaotong University, 277 Yanta West Road, Xi’an, Shaanxi Province 710061 China; 2 Department of Medical Oncology, The First Affiliated Hospital of Xi’an Jiaotong University, 277 Yanta West Road, Xi’an, Shaanxi Province 710061 China

**Keywords:** Cancer stem cells, Apoptosis, Cell growth

## Abstract

Hepatocellular carcinoma (HCC) is characterised by a hypoxic microenvironment and a high rate of heterogeneity and recurrence, and the presence of cancer stem cells (CSCs) in HCC may well explain both of these pathological properties. There is mounting evidence that long non-coding RNAs (lncRNAs) participate in carcinogenesis and maintain cancer stemness of HCC cells. However, the expression modes, regulatory mechanisms and potential roles of stemness-related lncRNAs in HCC are still obscure. LncRNA RUNX1-IT1 is the intronic transcript 1 of the RUNX1, which is also known as chromosome 21 open-reading frame 96 (C21orF96). Although the functions of the RUNX1 have been identified in different diseases, the function and its potential mechanisms of the lncRNA RUNX1-IT1 in HCC still remains to be largely unknown. In this study, we verified that the expression of LncRNA RUNX1-IT1 was decreased in GEO data set, HCC samples and correlated with unfavourable clinicopathologic characteristics and poor prognosis. RUNX1-IT1 repressed HCC cell proliferation, cell cycle progression, invasion and cancer stemness and induced apoptosis in vitro. Overexpression of RUNX1-IT1 impaired the growth, metastasis and stem-like features of HCC cells in vivo. Mechanistically, RUNX1-IT1 directly bound to miR-632 and acted as competing endogenous RNA to facilitate the expression of the miR-632 target gene GSK-3β and subsequently modulate the WNT/β-catenin pathway in HCC cells. Furthermore, hypoxia-driven histone deacetylase 3 (HDAC3), as an upstream regulatory mechanism, was critical for the downregulation of RUNX1-IT1 in HCC. Thus, lncRNA RUNX1-IT1, as a regulator of hypoxia, may function as a potential therapeutic target for conquering HCC.

## Introduction

Hepatocellular carcinoma (HCC) remains one of the most common malignancies and accounts for the third leading cause of cancer-associated deaths worldwide^1^. Epidemiological data revealed 841,000 new cases of liver cancer and 782,000 mortalities worldwide, among which China represents ~50% of the total number of new cases and deaths^2–4^. Despite continuous advancement in medical technology over the past decades, the 5-year survival rate of HCC patients is still <30% because of the high rate of recurrence and heterogeneity^5^. Thus, it is urgent to explore the detailed molecular mechanisms involved in HCC tumorigenicity and heterogeneity and discover more efficient treatment targets for HCC.

Epithelial–mesenchymal transition (EMT) is a cellular programme conferring an enhanced potential for cancer initiation and metastasis on tumour cells and promoting a greater resistance to chemotherapy^6^. HCC is characterised by a high rate of heterogeneity and recurrence, and cancer stem cells (CSCs) present in HCC may well explain both of these pathological properties. Although the molecular definition of CSCs in HCC is still emerging such as CD24, CD44, CD90 and EPCAM^7,8^, three transcription factors, Oct4, Nanog and Sox2, have been strongly identified as master regulators of cancer stemness^9,10^. Intriguingly, there exists a tight link between acquiring cancer stem-like traits and EMT induction^11^. However, the underlying mechanisms that contribute to the self-renewal and maintenance of CSCs are poorly understood.

Previous studies have commonly concentrated on the effects of the protein-coding genome on cancer stemness. Over the past decades, however, the understanding of the non-coding genome and its influence on cell fates has greatly expanded^12^. Stem cells provide an attractive system for investigating long non-coding RNAs (lncRNAs) effects as previous discoveries have uncovered that the expression mode of lncRNAs, identified as transcripts longer than 200 nucleotides, is more cell type-specific in comparison with mRNA expression^13^, resulting in the possibility that lncRNAs may be key modulators of stem-like features. For example, lncRNA BRM has been recognised to have vital roles in maintaining liver cancer stemness. With respect to its potential mechanisms, lncRNA BRM directly binds to BRM, resulting in a switch from BRM-embedded BAF to BRG1-embedded BAF, and then BRG1-embedded BAF modulates the self-renewal of liver CSCs via KLF4-dependent Yap activation^14^. Interestingly, the oncofetal lncRNA PVT1 has been illustrated to facilitate proliferation, promote cell cycle progression and markedly elevate the cancer stem-like traits of HCC cells by directly interacting with an RNA-binding protein named NOP2 and maintaining its stability^15^. In addition, the novel lncRNA DANCR, which can competitively bind to *CTNNB1* (β-catenin) to relieve the inhibitory effects of miR-199a, miR-320a and miR-214 on *CTNNB1*, markedly enhances the cancer stemness properties of HCC cells to confer tumorigenesis and intrahepatic colonisation or lung metastasis in HCC^16^. However, the explicit mechanism of action of diverse lncRNAs on the biology of hepatic CSCs remains largely obscure and needs to be elucidated urgently.

Almost all solid tumours exist in a hypoxic microenvironment, and hypoxia accelerates the malignant progression of cancers such as facilitating tumour proliferation, metastasis and cancer stemness^17^. Moreover, hypoxia/HIF-1α signalling has previously been shown to modulate the non-coding transcriptome including lncRNAs and miRNAs^18,19^. LncRNA RUNX1-IT1 is the intronic transcript 1 of RUNX1, which is also known as chromosome 21 open-reading frame 96 (C21orF96). Even though the roles of RUNX1 in different diseases, including in haematopoiesis^20^ and as a tumour suppressor in a variety of cancers, have been identified^21,22^, the function and potential mechanisms of lncRNA RUNX1-IT1 in HCC remain largely unknown. In this study, we verified that lncRNA RUNX1-IT1 restrained proliferation and cancer stem-like properties of HCC cells by acting as a molecular sponge for miR-632 to repress Wnt/β-catenin signalling. Moreover, hypoxia-driven histone deacetylase 3 (HDAC3) is critical for the downregulation of RUNX1-IT1 in HCC.

## Materials and methods

### Human HCC tissue samples

A total of 87 human HCC tissue samples and matched adjacent non-tumourous specimens were used to detect RUNX1-IT1 RNA levels, and miR-632 expression were collected from the First Affiliated Hospital of Xi’an Jiaotong University from January 2009 to December 2013. All samples were histopathologically confirmed, and the patients had not received any radiotherapy or chemotherapy before surgery. The collected specimens were stored at −80 °C. All procedures performed in studies involving human participants were in accordance with the ethical standards of the Research Ethics Committee of The First Affiliated Hospital of Xi’an Jiaotong University and with the 1964 Helsinki declaration and its later amendments. All written informed consent to participate in the study was obtained from HCC patients for samples to be collected from them.

### Cell culture

A human normal liver cell line (L02) and liver cancer cell lines (MHCC-97H, MHCC-97L, HepG2, Hep3B, Huh7, SK-HEP-1, PLC/PRF/5 and SMMC-7721) were obtained from the Cell Bank of the Chinese Academy of Sciences (Shanghai, China) and cultured in Dulbecco’s modified Eagle’s medium (Gibco, Grand Island, NY, USA) supplemented with 10% fetal bovine serum (Gibco, Grand Island, NY, USA) and 1% penicillin–streptomycin (Invitrogen, CA, USA). To assess the efficacy of hypoxia, cells were cultured under normoxic conditions to 60–70% confluence and then cultured under consistent 1% O_2_ hypoxic conditions for 24 or 48 h.

### Quantitative real-time PCR

We used TRIzol Reagent (Thermo Fisher Scientific, California, USA) to extract total RNA of HCC tissue specimens or HCC cell lines and quantitated the RNA concentration by absorbance at 260 nm. For mRNA detection, RNA samples (1 μg) were reverse transcribed using PrimeScript RT Master Mix, and quantitative real-time PCR was performed with SYBR-Green PCR Master Mix (Takara Bio, Dalian, China) using gene-specific primers. GAPDH was used as an endogenous control, and the results were calculated by the 2^−ΔΔCt^ method. To measure miRNA expression, mature miRNA-632 was reverse transcribed using a TaqMan MicroRNA Reverse Transcription Kit (Applied Biosystems, Foster City, CA, USA). U6 was utilised as an endogenous control to normalise miR-632 expression. The sequence of specific primers is displayed in Supplementary Table 1.

### Cytoplasmic and nuclear RNA isolation

Thermo Fisher BioReagents (Thermo Fisher Scientific) was utilised to extract nuclear and cytoplasmic RNAs or proteins following the manufacturer’s instructions. qRT-PCR analysis was conducted to detect the localisation of RUNX1-IT1 as previously reported^23^.

### RNA fluorescent in situ hybridisation

Subcellular localisation of RUNX1-IT1 was examined by the FISH Kit (RiboBio, Guangzhou, China) following the manufacturer’s instructions. In brief, MHCC-97H and HepG2 cells were plated on glass coverslips into 24-well plates. Subsequently, the cells were fixed in 4% paraformaldehyde for 15 min at room temperature. Then, the fixed cells were rinsed with phosphate-buffered saline (PBS) and underwent permeabilization (0.5% Triton X PBS for 15 min). After that, the cells were incubated with prehybridization solution and hybridised using hybridisation solution and then incubated with Cy3-labelled RUNX1-IT1 oligonucleotide probe (obtained from RiboBio, Guangzhou, China) overnight. Cell nuclei were stained with 4,6-diamidino-2-phenylindole for 5 min at room temperature. Images were obtained and recorded with appropriate excitation and emission spectra at a magnification of ×400 by a Zeiss Instruments confocal microscope (Zeiss, Oberkochen, Germany).

### Cell transfection

pcDNA3.1-control (vector) and pcDNA3.1-RUNX1-IT1 (RUNX1-IT1) were purchased from Shanghai GenePharma Co., Ltd. (Shanghai, China) as previously reported^24^. Scrambled shRNA (shControl) and RUNX1-IT1 shRNAs (shRNA#1 and shRNA#2) were obtained from Invitrogen (Carlsbad, CA, USA). MiR-632 inhibitors and miR-632 mimics were synthesised from Genecopoeia (Guangzhou, China). The siRNAs against GSK-3β, HIF-1α and HDAC3 were designed and synthesised by Shanghai GenePharma Co., Ltd. (Shanghai, China). The sequences were as follows: si-GSK-3β/sense 5′-CGAUUACACGUCUAGUAUA-3′ and si-GSK-3β/antisense 5′- UAACAAUCUAUUUACACCC-3′; si-HDAC3/sense 5′-UCGCCUGGCAUUGACCCAUTT-3′ and si-HDAC3/antisense 5′-AUGGGUCAAUGCCAGGCGATT-3′; si-HIF-1α/sense 5′-UGUGAGUUCGCAUCUUGAUTT-3′ and si-HIF-1α/antisense 5′-TTACACUCAAGCGUAGAACUA-3′. The GSK-3β Human cDNA ORF Clone (GSK-3β) was purchased from OriGene (OriGene Technologies, Inc., USA). Cell transfection was conducted as previously described^25^. Forty-eight hours post transfection, the cells were utilised for further experiments.

### Luciferase reporter assay and RNA immunoprecipitation (RIP) assay

The assays were performed according to previously described protocols^26^. In brief, for 3′-UTR luciferase reporter assays, the 3′-UTR of RUNX1-IT1 or GSK-3β was amplified by PCR and inserted downstream of the luciferase reporter gene in the pEZX-MT06 vector (Genecopoeia, Guangzhou, China). Point mutations of the miR-632-targeting sites in the RUNX1-IT1 or GSK-3β 3′-UTR were generated using the QuickChange Multiple Site-directed Mutagenesis Kit (Stratagene, La Jolla, CA). The method of construction with TCF/LEF1 reporter was as previously reported^16^. The designated vectors were co-transfected using Lipofectamine 2000 (Invitrogen, Carlsbad, CA, USA) into the indicated HCC cells cultured in 24-well plates. Renilla and firefly luciferase activities were detected after 48 h with the Luc-Pair Duo-Luciferase Assay Kit (Genecopoeia, Guangzhou, China). Renilla luciferase activity was normalised to firefly activity and represented relative luciferase activity. For the RIP assay, cells were collected and lysed in complete RIP lysis buffer. Then, the cell extract was incubated with RIP buffer containing magnetic beads conjugated to human anti-Ago2 antibody (Millipore, USA). Samples were incubated with proteinase K with shaking to digest proteins, and the immunoprecipitated RNA was purified. Subsequently, the isolated RNA was subjected to real-time PCR analysis.

### RNA pull-down assay

HCC cells were transfected with biotinylated wild-type (wt) miR-632, mutant (mut) miR-632 and negative control (NC) (Guangzhou RiboBio Co., Ltd). Forty-eight hours post transfection, cell lysates were harvested and incubated with Dynabeads M-280 Streptavidin (Invitrogen, CA, USA) at 4 °C for 3 h following the manufacturer’s protocol. Next, the beads were rinsed with ice-cold lysis buffer three times and washed once with high-salt buffer (0.1% SDS, 1% Triton X-100, 2 mM EDTA, 20 mM Tris-HCl, pH 8.0 and 500 mM NaCl)^27^. The bound RNAs were isolated and purified for subsequent qRT-PCR analysis.

### Determination of the ALDH^+^ cells, cell cycle, cell proliferation and cell apoptosis

The ALDH^+^ cells were detected by using the ALDEFLUOR kit (Stem Cell Technologies) following with the manufacturer’s instruction. The proportion of ALDH^+^ cells were determined by flow cytometry (BD FACS Canto II) and analysed by FlowJo software. After completing the designated intervention, HCC cells were subjected to the MTT assay, EdU assay, colony formation assay and flow cytometry analysis to detect cell viability, cell proliferation, apoptosis and cell cycle progression. The detailed protocols of the MTT assay, colony formation assay and flow cytometry analysis were displayed in our previous studies^9,28^. In brief, the EdU incorporation assay was conducted with an EdU kit (Roche, Indianapolis, IN, USA) following the manufacturer’s instructions. The cells were visualised using a Zeiss Instruments confocal microscope at a magnification of ×200 and quantified by counting at least five random fields.

### Transwell invasion assay

After completing the designated treatment, the invasion assay was conducted using Transwell chambers (BD Biosciences, Franklin Lakes, NJ). In brief, 1 × 10^5^ MHCC-97H and HepG2 cells were added to the upper chamber with a Matrigel-coated membrane. After 24 hours of incubation at 37 °C cells invaded to the lower surface of the membrane were fixed and dyed with crystal violet. The results were analysed by counting the dyed cells using optical microscopy (×100 magnification) in five randomly selected fields.

### Tumour sphere formation assay

After completing the designated intervention, HCC cells were plated at a density of 5000 cells per well in six-well ultra-low attachment plates (Corning, Corning, NY, USA) and then cultured with serum-free Dulbecco’s Modified Eagle Medium/F12 medium (Gibco) supplemented with 20 ng/mL human EGF, 1% B27 (Invitrogen, Carlsbad, CA, USA) and 20 ng/mL fibroblast growth factor. Subsequently, HCC cells were incubated at 37 °C with 5% CO_2_ for 14 days. A microscope (Nikon Instruments Inc.) was used to count the number and determine the diameter of the tumourspheres at a magnification of ×200.

### Western blot

Total proteins of MHCC-97H and HepG2 (1 × 10^6^) were extracted utilising RIPA Lysis Buffer (Beyotime, Guangzhou, China). The concentration of the proteins was measured by a BCA protein assay kit (Pierce, Rockford, USA). The detailed protocols for western blotting were based on previously described methods^23^, and the primary antibodies used in this study are displayed in Supplementary Table 2. The bands of the designated proteins were visualised by using chemiluminescence ECL reagents (Pierce, Rockford, IL), and the images of the bands were obtained by the ChemiDoc XRS imaging system (Bio-Rad, USA). Densitometry analysis was performed using Image-Pro Plus 6.0 (Media Cybernetics).

### Immunofluorescence staining

HCC cells were fixed with 4% paraformaldehyde and permeabilized using 0.3% Triton X-100 for 15 min. Then, the fixed cells were incubated with anti-CD44 primary antibody overnight. The secondary antibody was an Alexa Fluor-conjugated IgG (Invitrogen, Carlsbad, CA, USA). The cells on the slides were imaged and visualised with the appropriate excitation and emission spectra at a magnification of ×400 using a Zeiss Instruments confocal microscope (Zeiss, Oberkochen, Germany).

### In vivo tumorigenesis assays

All animal experiments were conducted based on protocols approved by the ethical committee of Xi’an Jiaotong University. For the subcutaneously implanted tumour assay, 1 × 10^6^ MHCC-97H cells infected with Lenti-RUNX1-IT1 or mock vector (purchased from Shanghai GenePharma Co., Ltd.) were resuspended in 100 μL PBS and subcutaneously injected into the left flanks of 4-week-old female BALB/c nude mice (six mice per group), and the nude mice were obtained and raised in the Animal Center at Medical College, Xi’an Jiaotong University. Tumour growth was monitored once a week, and the tumour volume was calculated by the following formula: *V* (tumour volume: mm^3^) = 0.5 × [*w* (width: mm)]^2^ × *L* (longer diameter: mm). The nude mice were killed after 28 days, and the tumour specimens were weighed, fixed and then stained by immunohistochemistry for histological analyses. The immunohistochemistry procedure was as previously reported^23^. The primary antibodies used for immunohistochemistry are displayed in Supplementary Table 2. In addition, 1 × 10^3^ MHCC-97H cells infected with Lenti-RUNX1-IT1 or mock vector were subcutaneously injected into female BALB/c nude mice (eight mice per group) to further evaluate the effects of RUNX1-IT1 on tumour initiation of HCC.

An in vivo orthotopic liver tumour model in nude mice was established to evaluate intrahepatic and lung metastasis^23^. In brief, 1 × 10^6^ MHCC-97H cells infected with Lenti-RUNX1-IT1 or mock vector were resuspended in 100 μL PBS and subcutaneously injected into the livers of nude mice. The mice were killed after 5 weeks, and their livers and lungs were dissected, fixed and then prepared for standard histological detection. The number of metastatic tumour nodules in the liver and lung were determined by Haemotoxylin and Eosin (H&E) staining.

### Statistical analysis

All data are displayed as the mean ± standard deviation (SD) of three independent experiments. Student’s *t* test or one-way analysis of variance (ANOVA) followed by the LSD post hoc test were conducted to compare the differences between two groups or more than two groups, respectively. The paired *t* test was utilised to compare RUNX1-IT1 and miR-632 levels in HCC and matched adjacent non-tumorous specimens. Survival curves were calculated using the Kaplan–Meier method, and the differences were assessed by a log-rank test. Pearson’s correlation analysis was used to determine the correlation between RUNX1-IT1 and miR-632 or HIF-1α. *P* value < 0.05 was considered statistically significant.

## Results

### RUNX1-IT1 is downregulated in HCC and correlates with poor prognosis

**Fig. 1:**
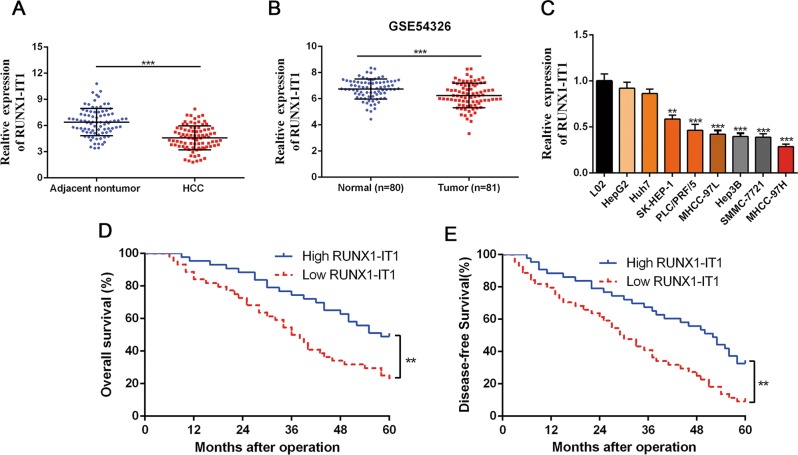
RUNX1-IT1 is a tumour suppressor with prognostic value. **a** The real-time PCR data from our patient’s cohort revealed that RUNX1-IT1 was significantly downregulated in HCC tissues (T, *n* = 87) than that in normal tissues (NT, *n* = 87). ***P < 0.001 by Student’s *t* test. **b** The GEO data set (GSE54236) from R2: Genomics Analysis and Visualisation Platform (http://r2.amc.nl) indicated that the expression of RUNX1-IT1 was prominently lower in HCC tissues compared with normal liver tissues. ***P < 0.001 by Student’s *t* test. **c** The expressions of RUNX1-IT1 in human normal hepatocyte cell line L02 and HCC cell lines SK-HEP-1, PLC/PRF/5, MHCC-97L, MHCC-97H, Huh7, SMMC-7721, HepG2 and Hep3B were examined using qRT-PCR. *n* = three independent experiments, **P < 0.01 or *** < 0.001 by Student’s *t* test versus LO2. **d**, **e** Kaplan–Meier survival curves of overall survival (OS) and disease-free survival (DFS) in our patients’ cohort. Patients were assigned into two subgroups according to the median expression of RUNX1-IT1. **P < 0.01 by two-sided log-rank test.

First, we utilised qRT-PCR to determine the expression pattern of RUNX1-IT1 in 87 HCC and matched adjacent non-malignant tissues. The results revealed that the expression level of RUNX1-IT1 in HCC specimens was greatly decreased in comparison with that of adjacent non-tumour tissues (*P* < 0.001, Fig. 1a). Furthermore, we analysed RUNX1-IT1 expression in a GEO data set (GSE54236), and in agreement with our data, RUNX1-IT1 was notably downregulated in HCC tissues (*P* < 0.001, Fig. 1b). In addition, RUNX1-IT1 expression was decreased in the HCC cell lines compared with the immortalised, normal human hepatic cell line L02 (*P* < 0.01, Fig. 1c). Analysis from clinical investigations indicated that the aberrant level of RUNX1-IT1 was closely correlated with clinicopathological parameters of HCC, including the number of tumour nodules, venous infiltration and Edmondson Steiner grading (*P* < 0.05, Supplementary Table 3). A strong association between low RUNX1-IT1 expression and shorter overall survival (OS) or disease-free survival (DFS) was observed when RUNX1-IT1 expression was compared in 87 HCC patients (*P* < 0.01, Fig. 1d, e). Collectively, these data suggest that downregulation of RUNX1-IT1 may lead to increased tumour aggressiveness.

### RUNX1-IT1 represses cell proliferation and the cell cycle and accelerates cell apoptosis in HCC

**Fig. 2:**
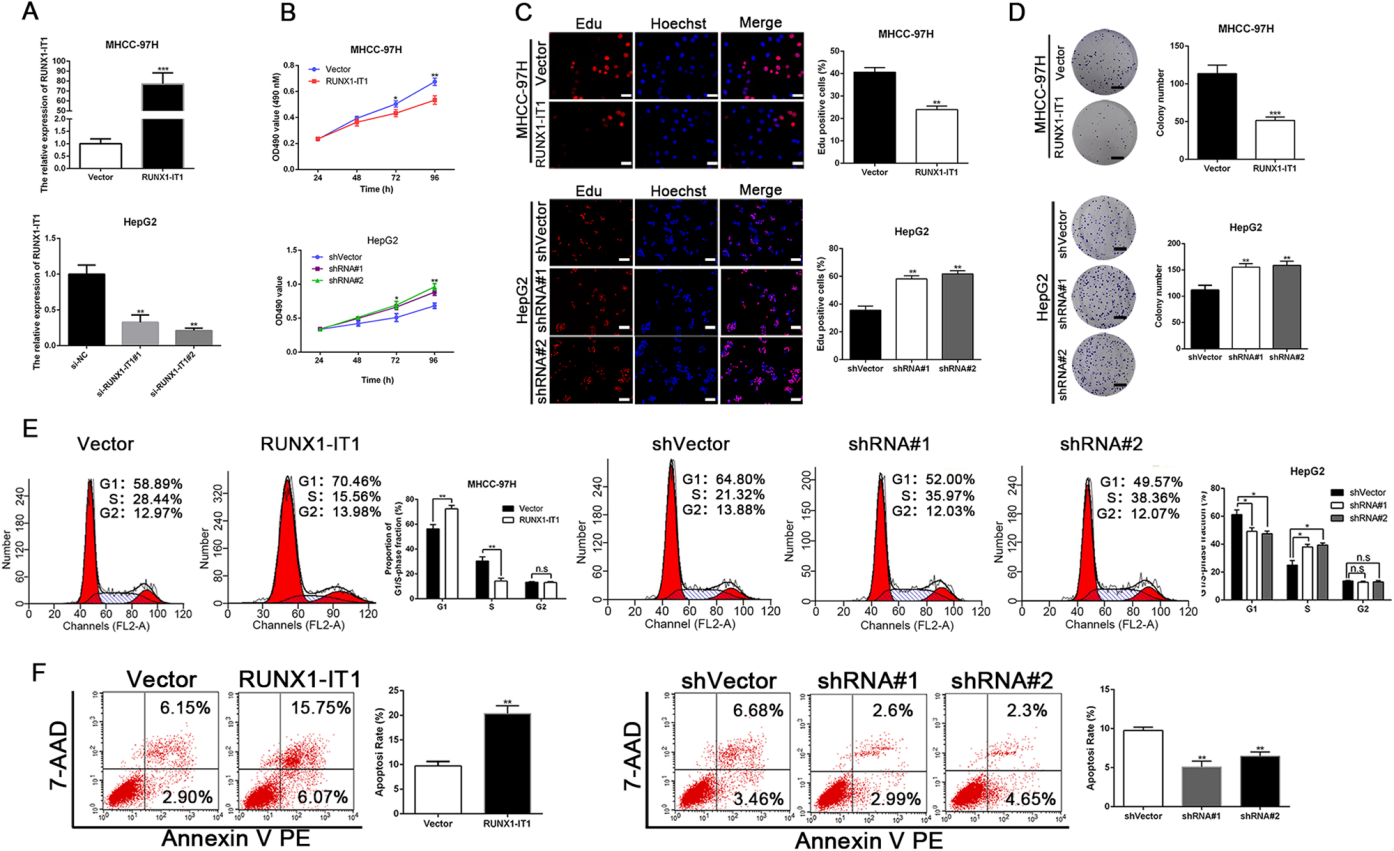
RUNX1-IT1 restrains HCC proliferation, cell cycle and facilitates cell apoptosis in vitro. **a** The expression level of RUNX1-IT1 in MHCC-97H cells was enhanced by pcDNA/RUNX1-IT1, and RUNX1-IT1 expression in HepG2 cells was reduced by sh-RUNX1-IT1 (shRNA#1, shRNA#2). *n* = three independent experiments, ***P < 0.001 by Student’s *t* test versus vector, or **P < 0.01 by ANOVA versus shVector. pcDNA/RUNX1-IT1 repressed whereas RUNX1-IT1 shRNA promoted cell viability **b**, cell proliferation **c**, colony formation **d** and cell cycle progression **e**. *n* = three independent experiments, *P < 0.05, **P < 0.01 or ***P < 0.001 by Student’s *t* test versus Vector or ANOVA versus shVector. **f** pcDNA/RUNX1-IT1 accelerated, whereas RUNXI-IT1 shRNA induced an inhibition of HCC cells apoptosis. *n* = three independent experiments, **P < 0.01 by Student’s *t* test versus Vector or ANOVA versus shVector. Magnification of Edu is ×200, and scale bars = 50 μm. The scale bars of colony formation = 1 cm.

To directly investigate the impact of RUNX1-IT1 on HCC cells, MHCC-97H cells were transfected with pcDNA/RUNX1-IT1, whereas HepG2 cells were transfected with RUNX1-IT1 shRNAs (shRNA#1 and shRNA#2). As displayed in Fig. 2a, real-time PCR analysis revealed that the level of RUNX1-IT1 was markedly modulated by pcDNA/RUNX1-IT1 and RUNX1-IT1-shRNAs in MHCC-97H and HepG2 cells, respectively (*P* < 0.01). Functionally, MTT, EdU, colony formation and flow cytometry assays were conducted to assess the effect of RUNX1-IT1 on cell proliferation and cell cycle progression in HCC. The results verified that overexpression of RUNX1-IT1 in MHCC-97H mitigated cell proliferation and induced cell cycle arrest, whereas RUNX1-IT1 depletion in HepG2 cells facilitated cell proliferation and cell cycle progression (*P* < 0.05, Fig. 2b–e). Moreover, flow cytometry analysis of apoptosis showed that pcDNA/RUNX1-IT1 increased the apoptosis of MHCC-97H cells (*P* < 0.01, Fig. 2f), whereas knockdown of RUNX1-IT1 in HepG2 cells had the opposite effect. Therefore, we conclude that RUNX1-IT1 represses cell proliferation, induces cell cycle arrest and increases apoptosis in HCC.

### Downregulation of RUNX1-IT1 induces EMT and confers CSC properties in HCC cells

**Fig. 3:**
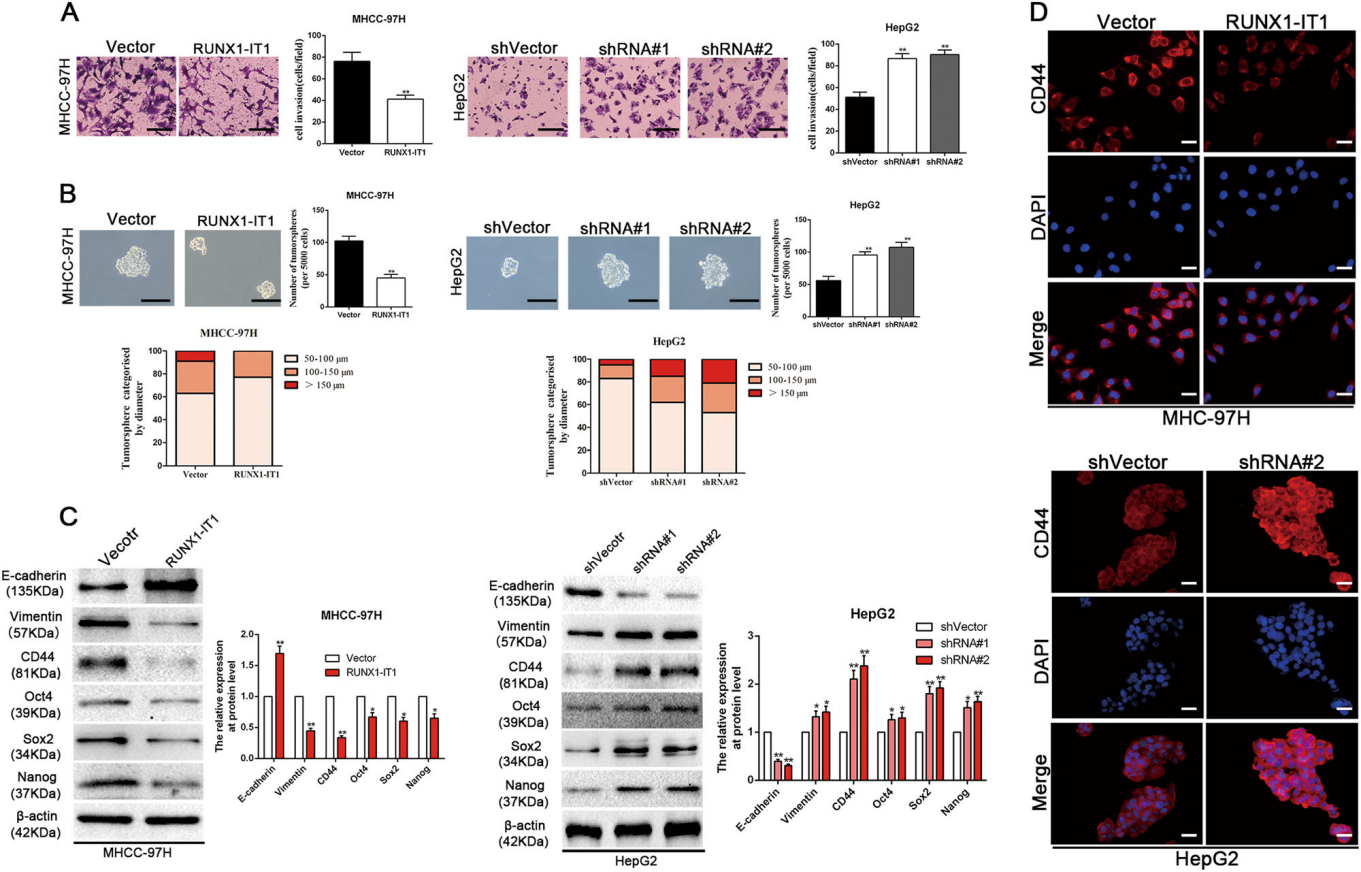
RUNX1-IT1 represses EMT and impedes cancer stemness in HCC cells. **a** Transwell assays showed that RUNX1-IT1 restoration reduced invasive abilities of MHCC-97H cells, whereas RUNX1-IT1 depletion enhanced the invasion of HepG2 cells. Scale bars = 50 μm. *n* = three independent experiments, **P < 0.01 by Student’s *t* test versus vector or ANOVA versus shVector. **b** Representative images of the tumorsphere formation assay were displayed after overexpression of RUNX1-IT1 in MHCC-97H cells and depletion of RUNX1-IT1 in HepG2 cells. The number of tumorspheres was counted and plotted, and the percentage of tumorspheres with diameters of 50–100 μm, 100–150 μm or >150 μm was calculated and plotted. Magnification is ×200, and scale bars = 50 μm. *n* = three independent experiments, **P < 0.01 by Student’s *t* test versus Vector or ANOVA versus shVector. **c** The protein expression levels of EMT and CSC markers after overexpression of RUNX1-IT1 in MHCC-97H cells and depletion of RUNX1-IT1 in HepG2 cells were determined by western blot analysis. β-Actin was used as an internal control. *n* = three independent experiments, *P < 0.05, **P < 0.01 by Student’s *t* test versus vector or ANOVA versus shVector. **d** Immunofluorescence analysis showed that RUNX1-IT1 restoration reduced CD44 expression in MHCC-97H cells, whereas RUNX1-IT1 depletion elevated the CCD44 expression in HepG2 cells. *n* = three independent experiments. Magnification is ×400, and scale bars = 20 μm.

Considering that EMT has an integral role in maintaining the cancer stem traits as well as metastatic progression of HCC, it is highly desirable to investigate the impacts of RUNX1-IT1 on EMT and cancer stemness of HCC. Intriguingly, RUNX1-IT1 overexpression in MHCC-97H cells repressed the invasive capacity and decreased the number and size of tumourspheres in comparison to MHCC-97H vector control cells (*P* < 0.01, Fig. 3a, b). The expression levels of EMT markers and several stemness genes, such as CD44, Sox2, Oct4 and Nanog, were significantly decreased by exogenous RUNX1-IT1 expression in MHCC-97H cells as confirmed by western blotting (*P* < 0.05, Fig. 3c). Subsequently, immunofluorescence analysis also displayed that enforced RUNX1-IT1 expression could result in decreased CD44 expression in MHCC-97H cells (Fig. 3d). In addition, RUNX1-IT1 overexpression can decrease the ALDH^+^ proportion of MHCC-97H cells, retard subcutaneous tumour formation derived from 1 × 10^3^ HCC cells and participate in therapeutic resistance (*P* < 0.05, Supplementary Fig 1). In contrast, RUNX1-IT1 depletion in HepG2 cells prominently induced EMT and conferred CSC properties in HCC cells. Together, these data indicated that RUNX1-IT1 could repress invasion, EMT and cancer stemness in HCC.

### RUNX1-IT1 represses tumorigenicity and the invasion-metastasis cascade in vivo

**Fig. 4:**
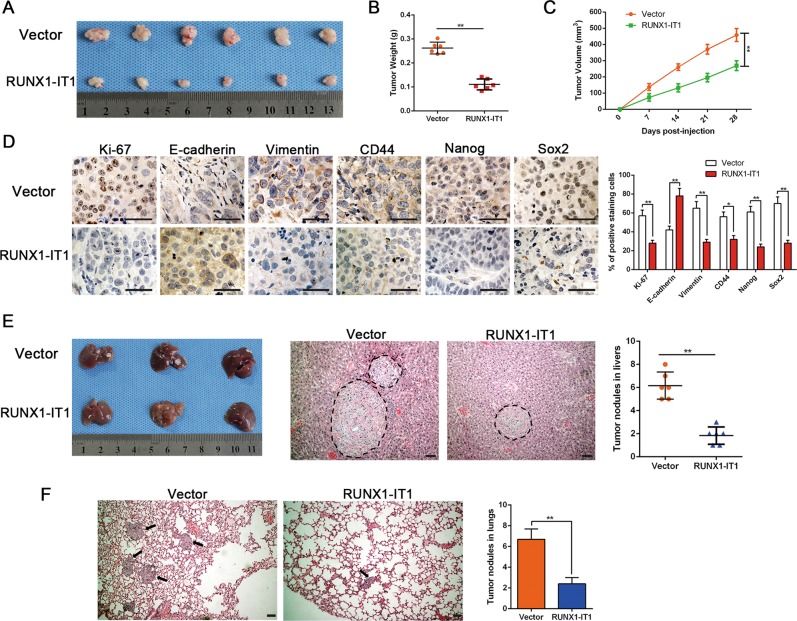
RUNX1-IT1 inhibits the tumorgenicity and metastasis of MHCC-97H cells along with reversing the mesenchymal and stem-like phenotypes. **a** Representative images of subcutaneous xenografts in nude mice implanted with MHCC-97H cells with Vector or overexpression RUNX1-IT1. (*n* = 6 per group). **b**, **c** Xenografts weight (mg) and tumour sizes were monitored and undergone quantification analysis. *n* = 6, **P < 0.01 by Student’s *t* test for tumour weight; **P < 0.01 by repeated-measures ANOVA for tumour sizes. **d** Immunohistochemistry staining and the semi-quantification analysis of Ki-67, E-cadherin, Vimentin, CD44, Nanog and Sox2 in xenograft tissues from different groups. Magnification is ×400, the scale bar represents 20 μm. *n* = 6, *P < 0.05 or **P < 0.01 by Student’s *t* test. **e** Representative images of orthotopic liver xenografts in nude mice implanted with MHCC-97H cells with Vector or overexpression RUNX1-IT1, representative hematoxylin and eosin–stained sections, and quantification analysis of tumour nodules in livers were displayed. Magnification is ×100, the scale bar represents 50 μm. *n* = 6, **P < 0.01 by Student’s *t* test. **f** Representative hematoxylin and eosin–stained sections, and quantification analysis of metastasis nodules in lungs were shown. Magnification is ×100, the scale bar represents 50 μm. *n* = 6, **P < 0.01 by Student’s *t* test.

To assess the efficacy of RUNX1-IT1 on the tumorigenicity of HCC cells in vivo, Lenti-RUNX1-IT1 and Lenti-vector MHCC-97H cells were subcutaneously injected into the flanks of nude mice to establish a xenograft tumour model. In vivo analyses of subcutaneous xenograft tumours of RUNX1-IT1 overexpression cells substantiated our in vitro findings. We observed that RUNX1-IT1 overexpression significantly inhibited HCC tumour growth compared with the vector control group (*P* < 0.01, Fig. 4a–c). Immunohistochemical analysis indicated that subcutaneous tumour tissues from the RUNX1-IT1 overexpression group exhibited weaker staining of Ki-67, vimentin and cancer stemness markers, including Sox2, Nanog, Oct4 and CD44, and stronger staining of E-cadherin compared with samples from the control group (*P* < 0.05, Fig. 4d). An emerging body of evidence has implicated the function of CSCs in tumour metastasis^29,30^. Therefore, we established an orthotopic liver tumour model in nude mice to explore the role of RUNX1-IT1 on intrahepatic diffusion and pulmonary colonisation of HCC. After the transplanted cells grew for 5 weeks, the nude mice were killed and the tumour nodules were detected. RUNX1-IT1 notably impeded the metastatic capacity of MHCC-97H cells in vivo. H&E staining of dissected livers and lungs from killed mice verified that fewer and smaller metastatic nodules were caused by RUNX1-IT1 overexpression (*P* < 0.01, Fig. 4e, f). Thus, these results offer in vivo support for our in vitro results that RUNX1-IT1 can effectively inhibit proliferation, EMT and stem-like traits in HCC cells.

### RUNX1-IT1 acts as a molecular sponge for miR-632 in HCC cells

**Fig. 5:**
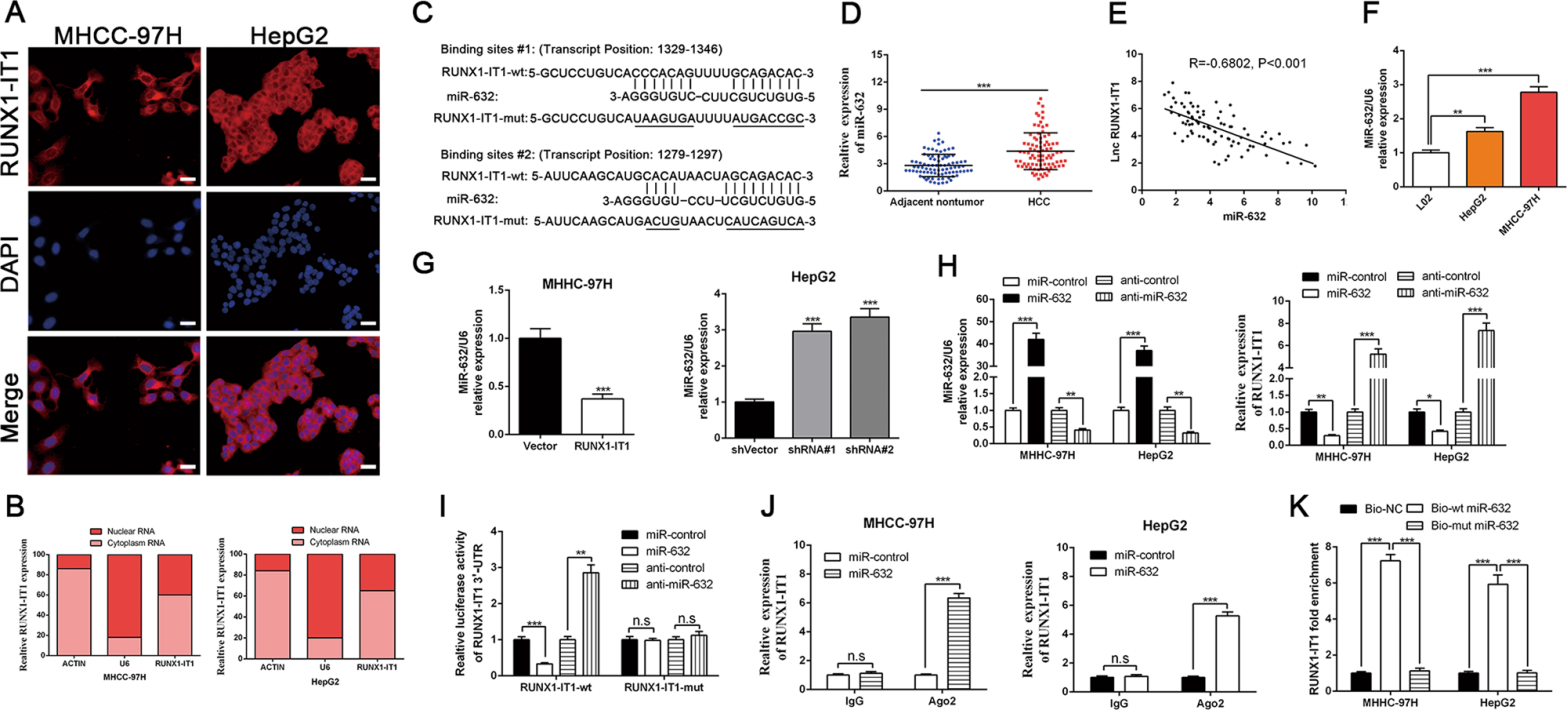
RUNX1-IT1 is abundant in cytoplasm and sponges miR-632 in HCC cells. RNA FISH assays **a** and subcellular fractionation assays **b** indicated that RUNX1-IT1 was predominately located in the cytoplasm. Magnification is ×400, and scale bars = 20 μm. **c** By applying bioinformatics tools (MIRDB, and DINAN tools-LncBase Predicted v.2), we found that there were two putative binding sites between 3′-UTR of RUNX1-IT1-wild type (wt) and miR-632. RUNX1-IT1-mutant (mut) means mutation of binding sites in the 3′-UTR of RUNX1-IT1. **d** The expression of miR-632 in tumour tissues (*n* = 87) was dramatically higher than that in adjacent non-tumour tissues (*n* = 87). ***P < 0.001 by Student’s *t* test. **e** Pearson correlation analysis verified that there existed a negative association between miR-632 and RUNX1-IT1 in HCC tissues. **f** MiR-632 was elevated in HCC cell lines. *n* = three independent experiments, **P < 0.01, ***P < 0.001 by Student’s *t* test versus L02. **g** Real-time PCR showed that miR-632 was negatively regulated by RUNX1-IT1. *n* = three independent experiments, ***P < 0.001 by Student’s *t* test versus vector or ANOVA versus shVector. **h** MiR-632 expression was greatly increased by miR-632 mimics, whereas significantly decreased by the inhibitors, and the expression of RUNX1-IT1 was negatively regulated by miR-632. *n* = three independent experiments, **P < 0.01, ***P < 0.001 by ANOVA. **i** Luciferase reporter gene assays showed that miR-632 negatively regulated the luciferase activity of RUNX1-IT1-wt-3′-UTR, rather than of RUNX1-IT1-mut-3′-UTR. *n* = three independent experiments, **P < 0.01, ***P < 0.001 by ANOVA. **j** The anti-Ago2 RIP assay with miR-632 mimics showed that both miR-632 and RUNX1-IT1 were enriched in Ago2 precipitate compared with IgG. *n* = three independent experiments. ***P < 0.001 by ANOVA. **k** RUNX1-IT1 was highly enriched in the sample pulled down by biotinylated wt miR-632 rather than mut miR-632. *n* = three independent experiments. ***P < 0.001 by ANOVA versus Bio-NC.

Next, we examined the potential molecular underpinnings by which RUNX1-IT1 inhibits EMT and cancer stem-like properties in HCC cells. RNA fluorescent in situ hybridisation and cell fractionation analysis were conducted to confirm the predominant cytoplasmic distribution of RUNX1-IT1 (Fig. 5a, b). The results indicated that RUNX1-IT1 is able to serve as a molecular sponge for miRNAs to regulate the malignant progression of HCC. Next, we aimed to identify the most likely miRNA that could be modulated by RUNX1-IT1 by utilising bioinformatics tools (miRDB: http://mirdb.org/ and DIANA tools-LncBase Predicted v.2). The data demonstrated that RUNX1-IT1 has two predicted miR-632-targeting sites (Fig. 5c), and the binding score was up to 0.999 in DIANA, suggesting a strong possibility of RUNX1-IT1 acting as a ceRNA. In addition, miR-632 has been recognised as an oncogene in HCC^31^. Thus, we focused on miR-632. Through qRT-PCR analysis of 87 paired HCC specimens and adjacent non-tumour tissues, we also confirmed that miR-632 was greatly upregulated in HCC (*P* < 0.001, Fig. 5d). The high level of miR-632 in HCC samples predicted the poor prognosis with reduced the OS and DFS (*P* < 0.01, Supplementary Fig 2). Analyses of RUNX1-IT1 and miR-632 expression displayed a prominent inverse correlation (Pearson’s correlation coefficient = −0.6802, *P* < 0.001, Fig. 5e), suggesting that RUNX1-IT1 was inversely associated with miR-632 expression. MiR-632 expression was also elevated in both MHCC-97H and HepG2 cells compared with L02 cells (*P* < 0.01, Fig. 5f). Moreover, miR-632 was negatively modulated by RUNX1-IT1 in MHCC-97H-pcDNA/RUNX1-IT1 and HepG2-sh-RUNX1-IT1 cells (*P* < 0.001, Fig. 5g). The level of miR-632 in tumour samples from mice injected with RUNX1-IT1-overexpressing cells was significantly lower than that in control mice (*P* < 0.05, Supplementary Fig. 3a). We then altered the expression levels of miR-632 and found that the expression of RUNX1-IT1 was modulated by miR-632 (*P* < 0.05, Fig. 5h). Intriguingly, the luciferase reporter assay revealed that miR-632 directly targeted the 3′-UTR of RUNX1-IT1-wt to negatively modulate the luciferase activity of RUNX1-IT1-wt-3′-UTR, but not the 3′-UTR of RUNX1-IT1-mut (*P* < 0.05, Fig. 5i). MicroRNAs are identified as binding their targets and resulting in translational repression or RNA degradation in an AGO2-dependent mode. To investigate whether RUNX1-IT1 was modulated by miR-632 in such a way, an anti-AGO2 RIP assay was conducted in MHCC-97H and HepG2 cells transiently overexpressing miR-632. Endogenous RUNX1-IT1 pull-down by AGO2 was specifically enriched in miR-632-transfected cells (*P* < 0.001, Fig. 5j), indicating that RUNX1-IT1 is a bona fide target of miR-632. Moreover, RUNX1-IT1 was pulled down by biotin-labelled miR-632, whereas mutagenesis of the binding sites of RUNX1-IT1 in miR-632 disrupted the interaction between RUNX1-IT1 and miR-632 (*P* < 0.001, Fig. 5k, and Supplementary Fig. 3b). In addition, miR-632 mediated the effect of RUNX1-IT1 on cell proliferation, EMT and cancer stem-like properties in HCC cells (*P* < 0.05, Supplementary Figs. 4 and 5).

### MiR-632 activates Wnt/β-catenin signalling by directly targeting GSK-3β in HCC cells

**Fig. 6:**
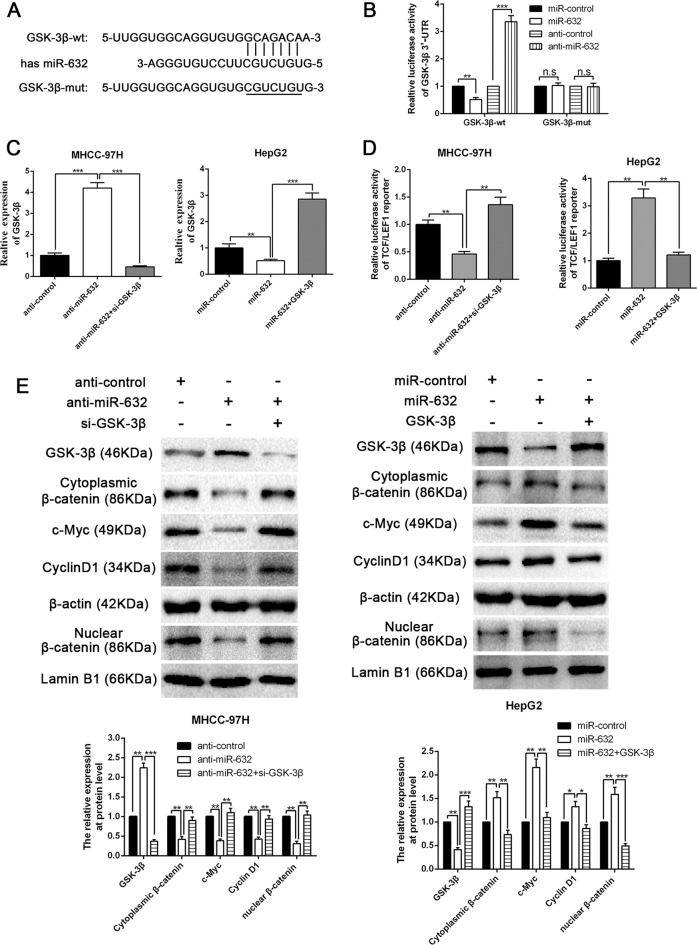
MiR-632 activates Wnt/β-catenin pathway by directly targeting GSK-3β. **a** Data from bioinformatics tools (microRNA.org, TargetScan and miRDB) showed that there were putative binding sites between 3′-UTR of GSK-3β-wt and miR-632. GSK-3β-mut means mutation of binding sites in 3′-UTR of GSK-3β. **b** Luciferase reporter gene assays illustrated that miR-632 negatively regulated the luciferase activity of GSK-3β-wt-3′-UTR, rather than of GSK-3β-mut-3′-UTR. *n* = three independent experiments. **P < 0.01, ***P < 0.001 by ANOVA. The mRNA **c** and protein **e** expression of GSK-3β was negatively modulated by miR-632, and GSK-3β clone or siRNAs reversed the effects of miR-632 mimics or inhibitors on GSK-3β expression. *n* = three independent experiments. **P < 0.01, ***P < 0.001 by ANOVA. **d** Luciferase activity of TCF/LEF1 reporters was regulated by modulation of miR-632 and GSK-3β. *n* = three independent experiments. **P < 0.01, by ANOVA. **e** Western blot results revealed that the accumulation of cytoplasmic β-catenin and the accumulation of nuclear β-catenin, c-Myc expression and cyclin D1 level were negatively modulated by miR-632, whereas reversed by GSK-3β clone and siRNA. *n* = three independent experiments. *P < 0.05, **P < 0.01 and ***P < 0.001 by ANOVA.

Interestingly, we found that the 3′-UTR of GSK-3β, which is known to inhibit the activation of the Wnt/β-catenin signalling pathway, harbours a putative binding site for miR-632 identified by using bioinformatics tools (microRNA.org, TargetScan and miRDB) (Fig. 6a). In addition, it has been illustrated that GSK-3β is a direct target of miR-632 in laryngeal carcinoma cells^32^. We investigated whether GSK-3β was a target of miR-632 in HCC cells. A luciferase reporter assay revealed that miR-632 negatively modulated the fluorescence intensity of GSK-3β-wt-3′-UTR but not that of GSK-3β-mut-3′-UTR (*P* < 0.01, Fig. 6b). The expression of GSK-3β was also negatively regulated by miR-632 in HCC cells (*P* < 0.01, Fig. 6c, e). The luciferase activity of TCF/LEF1 could be elevated by miR-632 overexpression and reduced by miR-632 inhibition, whereas its effect could be manipulated by simultaneously altering the GSK-3β level (*P* < 0.01, Fig. 6d). Moreover, the accumulation of cytoplasmic and nuclear β-catenin and the levels of downstream targets of the Wnt/β-catenin signalling pathway, such as cyclin D1 and c-Myc, in HepG2 cells were increased by miR-632 mimics (*P* < 0.05, Fig. 6e). On the other hand, miR-632 inhibitors had the opposite effect in MHCC-97H cells (*P* < 0.01, Fig. 6e). Conversely, GSK-3β partly reversed the effect of miR-632 on the expression of these proteins (*P* < 0.05, Fig. 6e). Therefore, we conclude that miR-632 directly targets GSK-3β to stimulate Wnt/β-catenin signalling in HCC cells.

### RUNX1-IT1 inhibits Wnt/β-catenin signalling by the RUNX1-IT1/miR-632/GSK-3β cascade

**Fig. 7:**
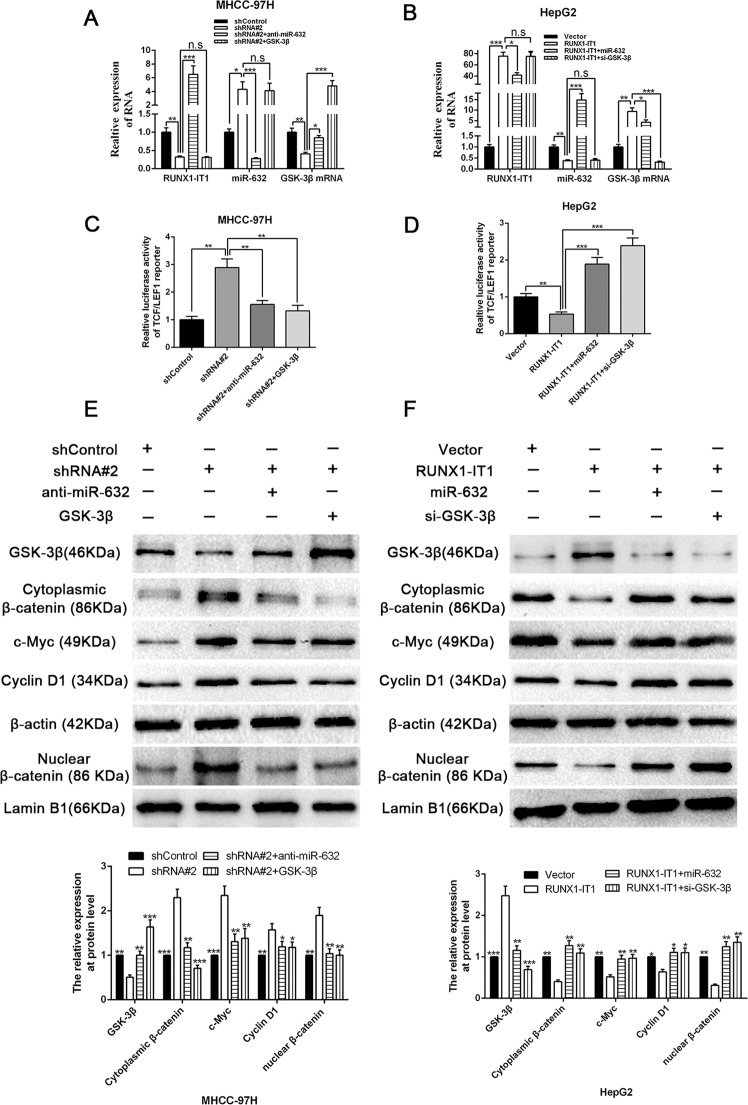
RUNX1-IT1 suppresses Wnt/β-catenin pathway through RUNX1-IT1/miR-632/ GSK-3β cascades. Rescue experiments revealed that RUNX1-IT1 negatively regulated the miR-632 expression **a**, **b**, whereas positively regulated both the mRNA **a**, **b** and protein **e**, **f** expression of GSK-3β by RUNX1-IT1/miR-632/GSK-3β cascades. *n* = three independent experiments. *P < 0.05, **P < 0.01 and ***P < 0.001 by ANOVA. **c**, **d** Luciferase activity of TCF/LEF1 reporters was regulated by modulation of RUNX1-IT1/miR-632/ GSK-3β axis. *n* = three independent experiments. **P < 0.01, ***P < 0.001 by ANOVA. **e**, **f** Western blot results from rescue experiments showed that RUNX1-IT1 negatively regulated the accumulation of cytoplasmic β-catenin and the accumulation of nuclear β-catenin, c-Myc expression and cyclin D1 level by RUNX1-IT1/miR-632/GSK-3β axis. *n* = three independent experiments. *P < 0.05, **P < 0.01 and ***P < 0.001 by ANOVA.

We performed rescue experiments and corroborated that RUNX1-IT1 positively modulated GSK-3β mRNA levels, which was mediated by miR-632 (*P* < 0.05, Fig. 7a, b). We then transfected the luciferase reporter plasmid TCF/LEF1 into MHCC-97H and HepG2 cells and discovered that the luciferase activity could be decreased by RUNX1-IT1 overexpression and enhanced by RUNX1-IT1 inhibition, whereas the effect of RUNX1-IT1 on luciferase activity could be manipulated by simultaneously altering the expression level of miR-632 or GSK-3β (*P* < 0.01, Fig. 7c, d). sh-RUNX1-IT1 mitigated the expression of GSK-3β but enhanced the accumulation of cytoplasmic and nuclear β-catenin and the expression of cyclin D1 and c-Myc, whereas miR-632 inhibitors or a GSK-3β clone (overexpression of GSK-3β) reversed the potency of sh-RUNX1-IT1 (*P* < 0.05, Fig. 7e). Moreover, miR-632 mimics or si-GSK-3β reversed the effects of pcDNA/RUNX1-IT1 in HepG2 cells (*P* < 0.05, Fig. 7f). The expression of β-catenin protein in tumour tissues from mice injected with RUNX1-IT1-overexpressing cells was notably lower than that in control mice (*P* < 0.01, Supplementary Fig. 6a, b). HCC tissues with high RUNX1-IT1 or low miR-632 levels displayed an obvious lower level of β-catenin protein in comparison with cases with low RUNX1-IT1 or high miR-632 level (*P* < 0.001, Supplementary Fig. 6c, d). We illustrated that direct manipulation of GSK-3β/β-catenin pathway could modulate cancer stemness (*P* < 0.05, Supplementary Fig. 7a, b), and also excluded the effects of Wnt on activation of GSK-3β/β-catenin pathway during this process (*P* < 0.01, supplementary Fig. 7c, d). In addition, GSK-3β can mediate the efficacy of RUNX1-IT1 on cell proliferation, EMT and cancer stem-like properties in HCC cells (*P* < 0.05, Supplementary Figs. 8 and 9).

### Hypoxia-driven HDAC3 is critical for downregulation of RUNX1-IT1 in HCC

**Fig. 8:**
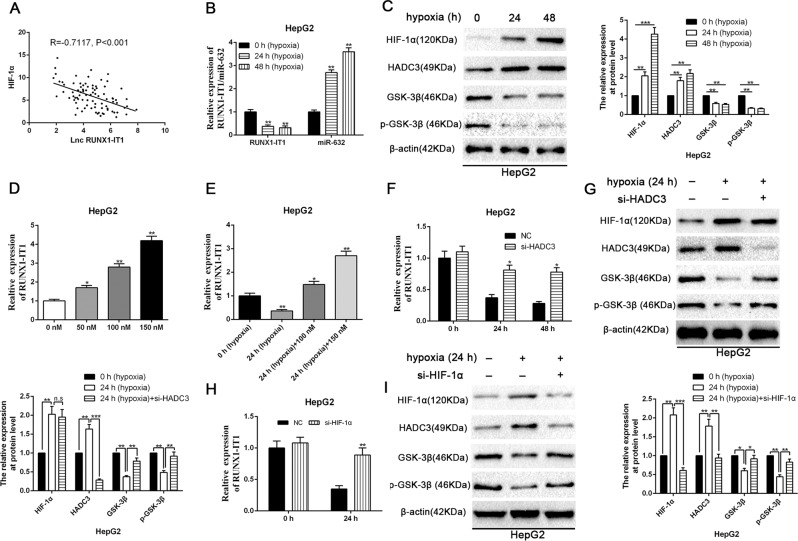
HDAC3 is critical to RUNX1-IT1 downregulation in HCC cells following hypoxia. **a** Pearson correlation analysis revealed that the expression levels of RUNX1-IT1 and HIF-1α were negatively correlated in 87 HCC samples, as measured by real-time PCR. **b** RUNX1-IT1 is downregulated, whereas miR-632 is upregulated under hypoxic culture conditions (1% O_2_, 5% CO_2_, 94% N_2_) in HepG2 cells. *n* = three independent experiments. **P < 0.01 by ANOVA. **c** Hypoxia increased HIF-1α and HDAC3 protein levels, whereas decreased GSK-3β and p-GSK-3β protein levels. *n* = three independent experiments. **P < 0.01, ***P < 0.001 by ANOVA. **d** HepG2 cells were stimulated with varying concentrations of the histone deacetylase inhibitor trichostatin A (TSA) for 24 h, and RUNX1-IT1 was upregulated by the TSA. *n* = three independent experiments. *P < 0.05, **P < 0.01 by ANOVA. **e** The repression of RUNX1-IT1 expression by hypoxia could be reversed by TSA. *n* = three independent experiments. *P < 0.05, **P < 0.01 by ANOVA. **f** siRNA silencing HDAC3 arrested the downregulation of RUNX1-IT1 in hypoxia-induced HepG2 cells. *n* = three independent experiments. *P < 0.05 by Student’s *t* test. **g** Immunoblotting analysis revealed that the expression of hypoxia increased HIF-1α and HDAC3 protein levels, but decreased GSK-3β and p-GSK-3β protein levels. siRNA targeting HDAC3 was not influenced the expression of HIF-1α while erased the inhibition of GSK-3β and p-GSK-3β under hypoxic conditions. *n* = three independent experiments. **P < 0.01, ***P < 0.001 by ANOVA. **h** qRT-PCR analysis displayed that siRNA targeting HIF-1α arrested the downregulation of RUNX1-IT1 in hypoxia-induced HepG2 cells. *n* = three independent experiments. **P < 0.01 by Student’s *t* test. **i** Western blot analysis showed that siRNA targeting HIF-1α arrested the upregulation of HDAC3 protein level, and offset the downregulation of GSK-3β and p-GSK-3β protein levels in hypoxia-induced HepG2 cells. *n* = three independent experiments. *P < 0.05, **P < 0.01 and ***P < 0.001 by ANOVA.

It is known that the hypoxic microenvironment exerts a pivotal role on multiple processes of the malignant progression of HCC. Thus, we were eager to determine whether hypoxia could cause downregulation of RUNX1-IT1 in HCC. A statistically significant inverse correlation was found between HIF-1α and RUNX1-IT1 transcripts (*n* = 87, *r* = −0.7117, *p* < 0.01 by Pearson’s correlation, Fig. 8a). Interestingly, we found that hypoxic conditions reduced RUNX1-IT1 levels while enhancing the expression level of miR-632 in HepG2 cells (*P* < 0.01, Fig. 8b). Moreover, HCC-associated lncRNAs can be modulated by inhibitors of histone deacetylases, and hypoxia can also contribute to the histone acetylation disorder^19^. Thus, we wondered whether hypoxia-driven histone acetylation disorder participates in the downregulation of RUNX1-IT1 in HCC. To corroborate this hypothesis, we utilised trichostatin A (TSA), a histone deacetylase inhibitor, to treat HepG2 cells. Interestingly, we confirmed that the expression of RUNX1-IT1 was elevated by TSA (*P* < 0.05, Fig. 8d), and the suppression of RUNX1-IT1 expression induced by hypoxia could also be reversed by TSA (*P* < 0.05, Fig. 8e). Indeed, previous studies have substantiated that hypoxia specifically enhanced the expression of HDAC3 at the protein level without affecting other HDACs^19,33^, and overexpression of HDAC3 reduced the phosphorylation of GSK-3β^34^. Our western blot data also confirmed that hypoxia increased HDAC3 and reduced p-GSK-3β at the protein level (*P* < 0.01, Fig. 8c). We further addressed whether the suppression of RUNX1-IT1 in the hypoxic microenvironment was mediated by HDAC3. The level of RUNX1-IT1 was remarkably reduced in negative control group after hypoxia intervention, whereas the level of RUNX1-IT1 did not change under hypoxic conditions in HepG2 cells transfected with the HDAC3 siRNAs (*P* < 0.05, Fig. 8f). Specific siRNAs^19^ for depleting HDAC3 were transfected into HepG2 cells, and transfection efficiency was validated by western blotting (*P* < 0.01, Fig. 8g). Furthermore, the phosphorylation level of GSK-3β was rescued in HDAC3 siRNA-transfected HepG2 cells under hypoxic conditions, as confirmed by western blot analysis (*P* < 0.01, Fig. 8g). We next explored whether the hypoxic repression of RUNX1-IT1 could be impeded by HIF-1α knockdown (*P* < 0.01, Fig. 8h). As displayed in Fig. 8i, the expression of RUNX1-IT1 was rescued when HIF-1α was depleted by siRNAs in a hypoxic microenvironment (*P* < 0.01). The downregulation of p-GSK-3β and upregulation of HDAC3 at the protein level could also be abrogated (*P* < 0.05, Fig. 8i). In addition, RUNX1-IT1/miR-632/GSK-3β cascades also participated in modulation of EMT and cancer stem traits of HCC cells under hypoxic conditions (*P* < 0.01, Supplementary Fig. 10).

## Discussion

There is mounting evidence that lncRNAs participate in both physiological and pathological processes, such as inactivation of X chromatin and carcinogenesis^35,36^. Moreover, lncRNAs have been shown to have a pivotal role in tumorigenicity and metastasiss^37^. Stem cells provide an attractive system for investigating lncRNA features since previous discoveries have shown that lncRNA expression is more cell type-specific compared with mRNA expression^13^, resulting in the possibility that lncRNAs may be key modulators of stem-like features. However, how lncRNAs modulate the stem-like features of liver cancer cells is still largely unknown. Therefore, it is crucial to uncover the mechanisms by which lncRNAs control CSC features to develop better diagnostic and therapeutic strategies. At present, several lncRNAs have been reported to maintain liver cancer stemness through entirely different mechanisms to activate Wnt/β-catenin signalling. LncTCF7 is located in the nuclei of HCC cells, and its expression level is highly elevated in CD13^+^CD133^+^ liver CSCs and HCC tumours. Moreover, lncTCF7 is required to sustain the stem-like phenotypes of HCC cells. Mechanistically, lncTCF7 could activate Wnt/β-catenin signalling by directly recruiting the SWI/SNF complex to the promoter of TCF7 to trigger its expression^38^. In addition, a novel lncRNA, DANCR, which can competitively bind to *CTNNB1* (β-catenin) to relieve the inhibitory effects induced by miR-199a, miR-320a and miR-214 on *CTNNB1* markedly enhances the cancer stemness properties of HCC cells to confer tumorigenesis and intrahepatic colonization or lung metastasis in HCC^16^. Herein, we also report the vital involvement of lncRNA RUNX1-IT1 in the repression of cancer stemness through modulating miR-632/GSK-3β/β-catenin cascades.

In the present study, we verified that lncRNA RUNX1-IT1 was mainly located in the cytoplasm and the expression of lncRNA RUNX1-IT1 was reduced in HCC samples and HCC cell lines, and the downregulation of RUNX1-IT1 in tumours was validated to be a predictor of worse survival in HCC patients. Therefore, our data provide insight on the potential of lncRNA RUNX1-IT1 as an attractive molecular target for risk prognostication of HCC. Furthermore, lncRNA RUNX1-IT1 impeded cell proliferation, suppressed cell invasion, promoted cell apoptosis and induced cell cycle arrest. These results are in accordance with previous findings in colon cancer^24^. In addition, lncRNA RUNX1-IT1 also repressed stem features and prevented EMT in HCC by acting as a molecular sponge for miR-632 to modulate the Wnt/β-catenin signalling pathway. Based on the above findings, manipulation of the lncRNA RUNX1-IT1/Wnt/β-catenin signalling pathway may yield a novel strategy for the treatment of HCC.

A hypoxic microenvironment exists in almost all solid tumours, and the contribution of the hypoxia-modulated gene networks to malignant progression is of great interest^39^. Nevertheless, the effects of lncRNAs on hypoxia-responsive gene networks remain largely elusive. In this study, we discovered that there was an inverse correlation between the level of HIF-1α and the expression of RUNX1-IT1 in primary HCC specimens, and the repressive expression of RUNX1-IT1 in HCC tissues was associated with HCC metastasis. Furthermore, we identified the upstream mechanism by which RUNX1-IT1 is downregulated in HCC. Our results showed that hypoxia-driven HDAC3 decreased the expression level of RUNX1-IT1 and reduced the phosphorylation of GSK-3β. However, the limitation of this study is that we did not verify the mechanism that how hypoxia-driven HDAC3 decreased the expression level of RUNX1-IT1. We inferred that hypoxia-driven HDAC3 may influence the histone acetylation level on the promoter region of RUNX1-IT1 to further modulate RUNX1-IT1 expression. It will be confirmed in our future research. In addition, hypoxia-induced EMT and conferred cancer stem traits, which could be blocked by RUNX1-IT1 overexpression. Thus, lncRNA RUNX1-IT1, as a modulator of hypoxia, may function as a potential therapeutic target for conquering HCC.

## Supplementary information

Supplementary Figure legends

Supplementary Table 1

Supplementary Table 2

Supplementary Table 3

Supplementary Figure 1

Supplementary Figure 2

Supplementary Figure 3

Supplementary Figure 4

Supplementary Figure 5

Supplementary Figure 6

Supplementary Figure 7

Supplementary Figure 8

Supplementary Figure 9

Supplementary Figure 10
