# Supplementary material for: LncRNA RUNX1-IT1 which is downregulated by hypoxia-driven histone deacetylase 3 represses proliferation and cancer stem-like properties in hepatocellular carcinoma cells
Source: Cell Death Dis. 2020 Feb 5;11(2):95. doi: 10.1038/s41419-020-2274-x (PMC7002583; doi:10.1038/s41419-020-2274-x)
Supplement: Supplementary file 1 — Supplementary Figure legends [file 41419_2020_2274_MOESM1_ESM.docx]

**Supplementary Figure 1. Modulation the level of RUNX1-IT1 can influence cancer stemness of HCC cells.** (A) Flow cytometric analysis displayed that ALDH+ cell population decreased significantly after overexpression of RUNX1-IT1 in MHCC-97H cells. n =three independent experiments. **P < 0.01 by Student’s t-test. (B) The expression of RUNX1-IT1 was reduced after treatment with oxaliplatin in HepG2 and MHCC-97H cells. n =three independent experiments. *P < 0.05 by Student’s t-test. (C-D) RUNX1-IT1 overexpression had synergistic effect with chemotherapeutic drug: oxaliplatin by repressing MHCC-97H cells growth, while RUNX1-IT1 depletion in HepG2 cells had the opposite effects in HepG2 cells. n =three independent experiments. *P < 0.05, **P < 0.01 by ANOVA. (E) Overexpression of RUNX1-IT1 in MHCC-97H cells (1000 cells) can significantly suppress the tumor initiation in subcutaneous tumor formation model.

**Supplementary Figure 2. The prognosis of miR-632 in HCC patients.** The high level of miR-632 in HCC samples predicted the poor prognosis with reduced the OS(A) and DFS(B). **P < 0.01by two-sided log-rank test.

**Supplementary Figure 3.** (A) Xenograft tissues arising from RUNX1-IT1 overexpression group (n = 6) and control group (n = 6) were subjected to qRT-PCR for miR-632 expression. n = 6, *P < 0.05 by Student’s t-test. (B) The two putative binding sites between 3′UTR of RUNX1-IT1-wild type and miR-632 (wt) were analyzed by bioinformatics tools (microRNA.org, and DINAN tools-LncBase Predicted v.2). The sequences for mut forms of miR-632 were also shown.

**Supplementary Figure 4. The functions of RUNX1-IT1 are mediated by miR-632.** MiR-632 mimics promoted, whereas miR-632 inhibitors repressed HCC cells^,^ proliferation (A-C), cell cycle (E), cells^,^ invasion (F) and cells^,^ tumorsphere formation (G). Meanwhile, miR-632 mimics inhibited, whereas miR-632 inhibitors induced HCC cells' apoptosis (D). miR-632 mimics remarkably erased the inhibitory efficacy of RUNX1-IT1 on MHCC-97H cells^,^ proliferation (A-C), cell cycle (E), cells^,^ invasion (F) and cells^,^ tumorsphere formation (G), while reversed the promotion effects of RUNX1-IT1 on cells' apoptosis (D). miR-632 inhibitors reversed the promotion effects of sh-RUNX1-IT1 on HepG2 cells^,^ proliferation (A-C), cell cycle (E), cells^,^ invasion (F) and cells^,^ tumorsphere formation (G), while receded the inhibitory effects of sh-RUNX1-IT1 on cells' apoptosis (e). n =three independent experiments. *P < 0.05, **P < 0.01 by ANOVA.

**Supplementary Figure 5. The efficacy of RUNX1-IT1 on EMT and cancer stemness is mediated by miR-632.** MiR-632 mimics promoted, whereas miR-632 inhibitors repressed HCC cells^,^ EMT and cancer stemness (A-B). miR-632 mimics partially offset the inhibitory efficacy of RUNX1-IT1 on MHCC-97H cells^,^ EMT and cancer stemness (A). miR-632 inhibitors reversed the promotion effects of sh-RUNX1-IT1 on HepG2 cells^,^ EMT and cancer stemness (B). n =three independent experiments. *P < 0.05, **P < 0.01 by ANOVA.

**Supplementary Figure 6. The expression of miR-632 and β-catenin in xenograft tissues, and the relationships among RUNX1-IT1, miR-632 and β-catenin expression in HCC.** (A) Xenograft tissues arising from RUNX1-IT1 overexpression group (n = 6) and control group (n = 6) were subjected to immumohistochemical staining for detecting the expression of β-catenin. n = 6, **P < 0.01 by Student’s t-test. (B) Xenograft tissues arising from RUNX1-IT1 overexpression group (n = 6) and control group (n = 6) were subjected to immunoblotting for β-catenin protein expression, respectively. n = 6, **P < 0.01 by Student’s t-test. (C) Immunoblotting analysis revealed that the expression of β-catenin protein in HCC tissues with low miR-632 level (n = 43) was significantly lower than that in HCC tissues with high miR-632 level (n = 44). ***P < 0.001 by Student’s t-test. (D) Immunoblotting analysis revealed that the expression of β-catenin protein in HCC tissues with high RUNX1-IT1 level (n = 43) was significantly lower than that in HCC tissues with low RUNX1-IT1 level (n = 44). ***P < 0.001 by Student’s t-test.

**Supplementary Figure 7. The GSK-3β/β-Catenin signaling pathway directly modulated the cancer stem-like traits of HCC cells.** (A) Overexpression of GSK-3β in MHCC-97H cells repressed while depletion of GSK-3β in HepG2 cells facilitated the expression of surface maker: CD44 and stemness transcription factors：Oct4, Sox2 and Nanog. n =three independent experiments. *P < 0.05, **P < 0.01 by Student’s t-test. (B) Blockage of the Wnt/β-catenin pathway by XAV-939 attenuated while activation of the Wnt/β-catenin pathway by CHIR-99021elevated the expression of surface maker: CD44 and stemness transcription factors：Oct4, Sox2 and Nanog in MHCC-97H and HepG2 cells. n =three independent experiments. *P < 0.05, **P < 0.01 by Student’s t-test. (C) RUNX1-IT1 had no influence on the expression of WNT ligands：WNT3A, WNT5A. (D) Overexpression of RUNX1-IT1 can reverse the effect of WNT3A on the activation of β-Catenin. n =three independent experiments. **P < 0.01 by ANOVA.

**Supplementary Figure 8. Modulation the level of GSK-3β attenuates the role of RUNX1-IT1 in HCC cells.** MHCC-97H cells with RUNX1-IT1 overexpression (OE-RUNX1-IT1) were transfected with si-GSK-3β, and HepG2 cells with RUNX1-IT1 knockdown were transfected with GSK-3β vector. (A) MTT assay, (B) EdU incorporation assay, (C) colony formation, (D) apoptosis assay, (E) cell cycle assay, (F) Transwell-invasion assay and (G) tumorsphere formation assay were performed to measure cell proliferation, apoptosis, cell cycle progression, cell invasion and cancer stem-like phenotypes. n =three independent experiments. *P < 0.05, **P < 0.01 by ANOVA.

**Supplementary Figure 9. Modulation the level of GSK-3β attenuates the efficacy of RUNX1-IT1 on EMT and cancer stemness^,^ markers expression in HCC cells.** Depletion of GSK-3β rescued the RUNX1-IT1-induced inhibition in the HCC EMT and cancer stemness (A), on the other hand, restoration of GSK-3β remarkably abolished the promoting effects of RUNX1-IT1 knockdown on HCC cell EMT and cancer stemness (B). n =three independent experiments. *P < 0.05, **P < 0.01 by ANOVA.

**Supplementary Figure 10. Hypoxia-induced EMT and stem-like traits are modulated by RUNX1-IT1/miR-632/GSK-3β axis.** Hypoxia can induce invasion (A) and tumorsphere formation (B) in HepG2 cells. The abilities of invasion and tumorsphere formation in HepG2 cells in hypoxic conditions can be blocked by RUNX1-IT1 overexpression. The RUNX1-IT1-blocked invasion and tumorsphere formation abilities in HepG2 cells under hypoxic conditions were rescued by miR-632 overexpression or GSK-3β depletion. n =three independent experiments. **P < 0.01 by ANOVA. (C) hypoxia can induce EMT with downregulation of E-cadherin and upregulation of Vimentin, and promote the expression of cancer stemness markers with enhanced CD44, Oct4, Sox2 and Nanog levels. The RUNX1-IT1-blocked EMT and cancer stemness in HepG2 cells under hypoxic conditions were rescued by miR-632 overexpression or GSK-3β depletion. n =three independent experiments. *P < 0.05, **P < 0.01 by ANOVA.
