# Supplementary material for: LncRNA RUNX1-IT1 which is downregulated by hypoxia-driven histone deacetylase 3 represses proliferation and cancer stem-like properties in hepatocellular carcinoma cells
Source: Cell Death Dis. 2020 Feb 5;11(2):95. doi: 10.1038/s41419-020-2274-x (PMC7002583; doi:10.1038/s41419-020-2274-x)
Supplement: Supplementary file 2 — Supplementary Table 1 [file 41419_2020_2274_MOESM2_ESM.docx]

**Supplementary Table1.** Primers sequences for real-time PCR analysis

| **Gene** | **Primer Sequences** |
| --- | --- |
| RUNX1-IT1 | Forward: 5′-GGACACGCAGAGGAAGTCAA-3′ |
|  | Reverse: 5′-GTTCTTGAGGTTGGC GGAGA-3′ |
| MiR-632 | Forward: 5′-GACGGGAGGCGGAGCGGGGA-3′ |
|  | Reverse: 5′-TCCCCGCTCCGCCTCCCGTC-3′ |
| WNT3A | Forward: 5′-GACTTCCTCAAGGACAAGTACG-3′ |
|  | Reverse: 5′-GGCACCTTGAAGTAGGTGTAG-3′ |
| WNT5A | Forward: 5′-CCTTCGCCCAGGTTGTAAT-3′ |
|  | Reverse: 5′-AGAGAGGCTGTGCTCCTATAA-3′ |
| U6 | Forward: 5′-CTCGCTTCGGCAGCACA-3′ |
|  | Reverse: 5′-AACGCTTCACGAATTTGCGT-3′ |
| GAPDH | Forward: 5′-ACCACAGTCCATGCCATCAC-3′ |
|  | Reverse: 5′-TCCACCACCCTGTTGCTGAT-3′ |
