# Supplementary material for: LncRNA RUNX1-IT1 which is downregulated by hypoxia-driven histone deacetylase 3 represses proliferation and cancer stem-like properties in hepatocellular carcinoma cells
Source: Cell Death Dis. 2020 Feb 5;11(2):95. doi: 10.1038/s41419-020-2274-x (PMC7002583; doi:10.1038/s41419-020-2274-x)
Supplement: Supplementary file 3 — Supplementary Table 2 [file 41419_2020_2274_MOESM3_ESM.docx]

**Supplementary Table 2.** A list of the utilized primary antibodies

| **Antibody** | **Dilution & Use** | **Company** |
| --- | --- | --- |
| Rabbit anti-E-cadherin | 1:1000 (WB) | Cell Signaling Technology |
| Rabbit anti-β-catenin | 1:1000 (WB) | Cell Signaling Technology |
|  | 1:400 (IHC) |  |
| Rabbit anti-vimentin | 1:1000 (WB) | Cell Signaling Technology |
| Rabbit anti-CD44 | 1:1000 (WB) | Abcam |
|  | 1:300 (IF) |  |
| Rabbit anti-Sox2 | 1:1000 (WB) | Abcam |
| Rabbit anti-Oct4 | 1:1000 (WB) | Abcam |
| Rabbit anti-Nanog | 1:1000 (WB) | Abcam |
| Rabbit anti-ki-67 | 1:500 (IHC) | Abcam |
| Rabbit anti-Cyclin D1 | 1:2000 (WB) | Abcam |
| Rabbit anti-c-Myc | 1:1000 (WB) | Abcam |
| Rabbit anti-Lamin B1 | 1:1000 (WB) | Abcam |
| Rabbit anti-GSK-3β | 1:1000 (WB) | Abcam |
| Rabbit anti-p-GSK-3β | 1:1000 (WB) | Abcam |
| Rabbit anti-HDAC3 | 1:1000 (WB) | Abcam |
| Rabbit anti-HIF-1α | 1:1000 (WB) | Abcam |
| Goat anti-rabbit IgG-HRP | 1:10,000 (WB) | Abbkine. Inc |
| Goat anti-mouse IgG-HRP | 1:10,000 (WB) | Abbkine. Inc |
| Goat anti-Rabbit dylight 594  (red) IgG antibody | 1:150 (IF) | Abbkine. Inc |
