# Supplementary material for: LncRNA RUNX1-IT1 which is downregulated by hypoxia-driven histone deacetylase 3 represses proliferation and cancer stem-like properties in hepatocellular carcinoma cells
Source: Cell Death Dis. 2020 Feb 5;11(2):95. doi: 10.1038/s41419-020-2274-x (PMC7002583; doi:10.1038/s41419-020-2274-x)
Supplement: Supplementary file 4 — Supplementary Table 3 [file 41419_2020_2274_MOESM4_ESM.docx]

**Supplementary Table 3.** Correlation between RUNX1-IT1 expression and the clinicopathologic characteristics of hepatocellular carcinoma

| Characteristics | | Cases (n=87) | Number of patients | | *P* Value |
| --- | --- | --- | --- | --- | --- |
|  |  |  | RUNX1-IT1^high^ (n=43) | RUNX1-IT1^low^ (n=44) |  |
| Age (years) | <60 | 46 | 27 | 19 | 0.168 |
|  | ≥60 | 41 | 18 | 23 |  |
| Gender | Male | 73 | 40 | 33 | 0.871 |
|  | Female | 14 | 8 | 6 |  |
| HBV | Absent | 19 | 9 | 10 | 0.510 |
|  | Present | 68 | 38 | 30 |  |
| Serum AFP level (ng/ml) | <200 | 45 | 24 | 21 | 0.594 |
|  | ≥200 | 42 | 20 | 22 |  |
| Tumor size (cm) | <5 | 33 | 16 | 17 | 0.761 |
|  | ≥5 | 54 | 28 | 26 |  |
| Number of tumor nodules | 1 | 64 | 46 | 18 | 0.015* |
|  | ≥2 | 23 | 10 | 13 |  |
| Venous infiltration | Absent | 48 | 35 | 13 | 0.021* |
|  | Present | 39 | 19 | 20 |  |
| Edmondson-Steiner grading | Ⅰ+Ⅱ | 65 | 43 | 22 | 0.037* |
|  | Ⅲ+Ⅳ | 22 | 9 | 13 |  |
| TNM stage | Ⅰ+Ⅱ | 60 | 27 | 33 | 0.554 |
|  | Ⅲ+Ⅳ | 27 | 14 | 13 |  |

^*^Statistically significant.
