# Supplementary figures and images for: LncRNA RUNX1-IT1 which is downregulated by hypoxia-driven histone deacetylase 3 represses proliferation and cancer stem-like properties in hepatocellular carcinoma cells
Source: Cell Death Dis. 2020 Feb 5;11(2):95. doi: 10.1038/s41419-020-2274-x (PMC7002583; doi:10.1038/s41419-020-2274-x)

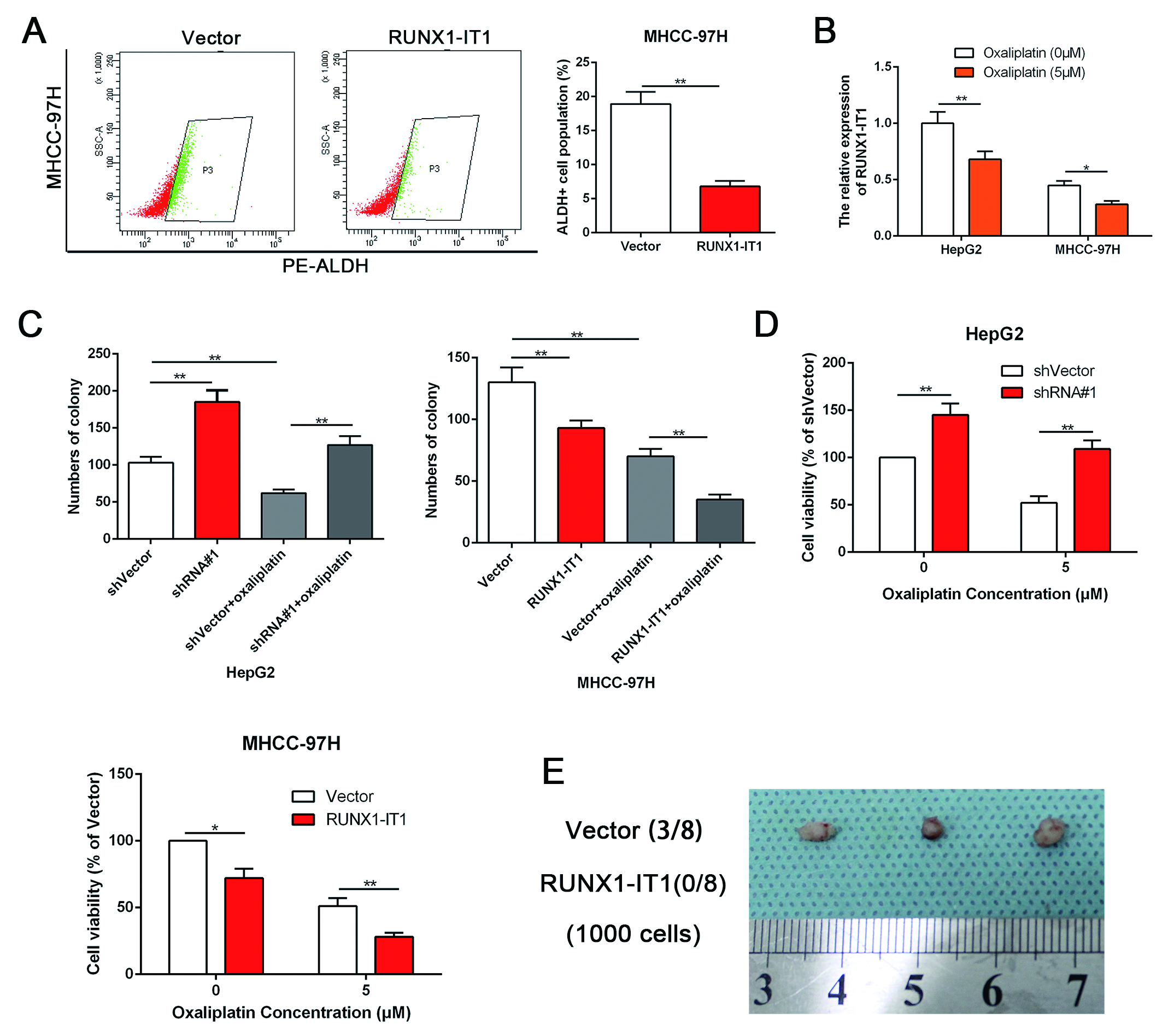

Supplement: Supplementary file 5 — Supplementary Figure 1 [file 41419_2020_2274_MOESM5_ESM.tif]

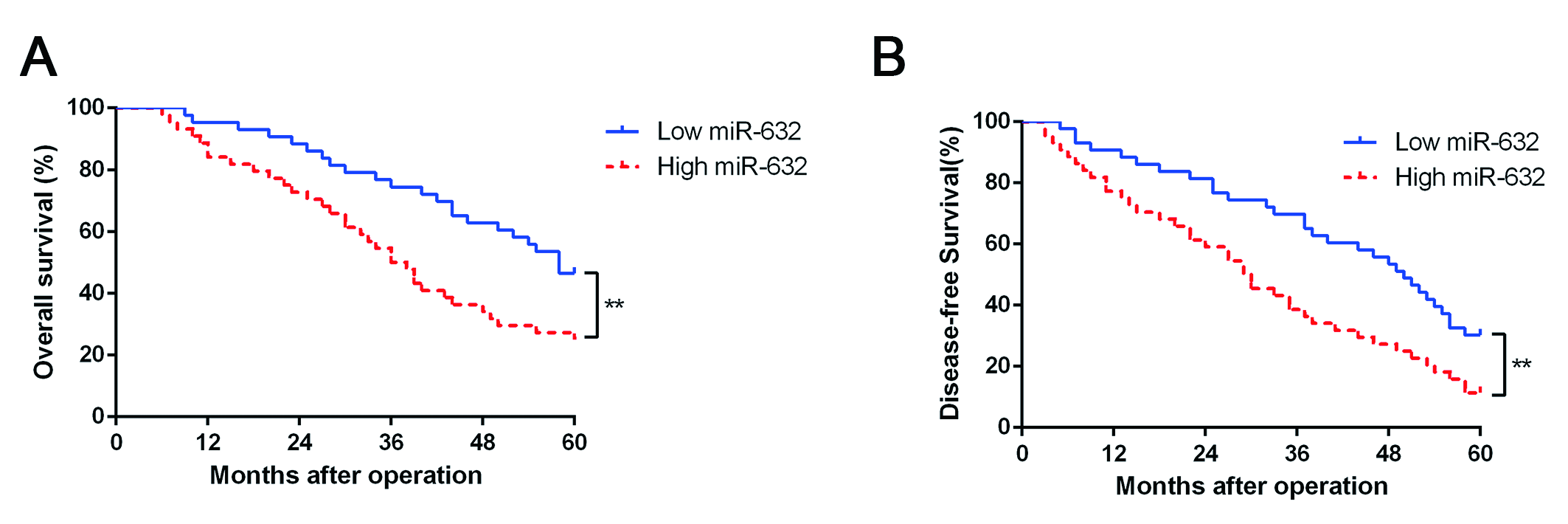

Supplement: Supplementary file 6 — Supplementary Figure 2 [file 41419_2020_2274_MOESM6_ESM.tif]

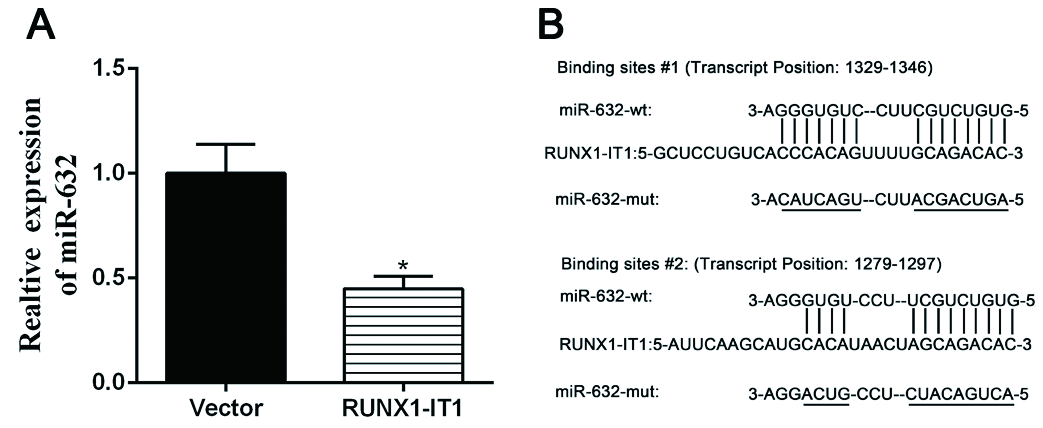

Supplement: Supplementary file 7 — Supplementary Figure 3 [file 41419_2020_2274_MOESM7_ESM.tif]

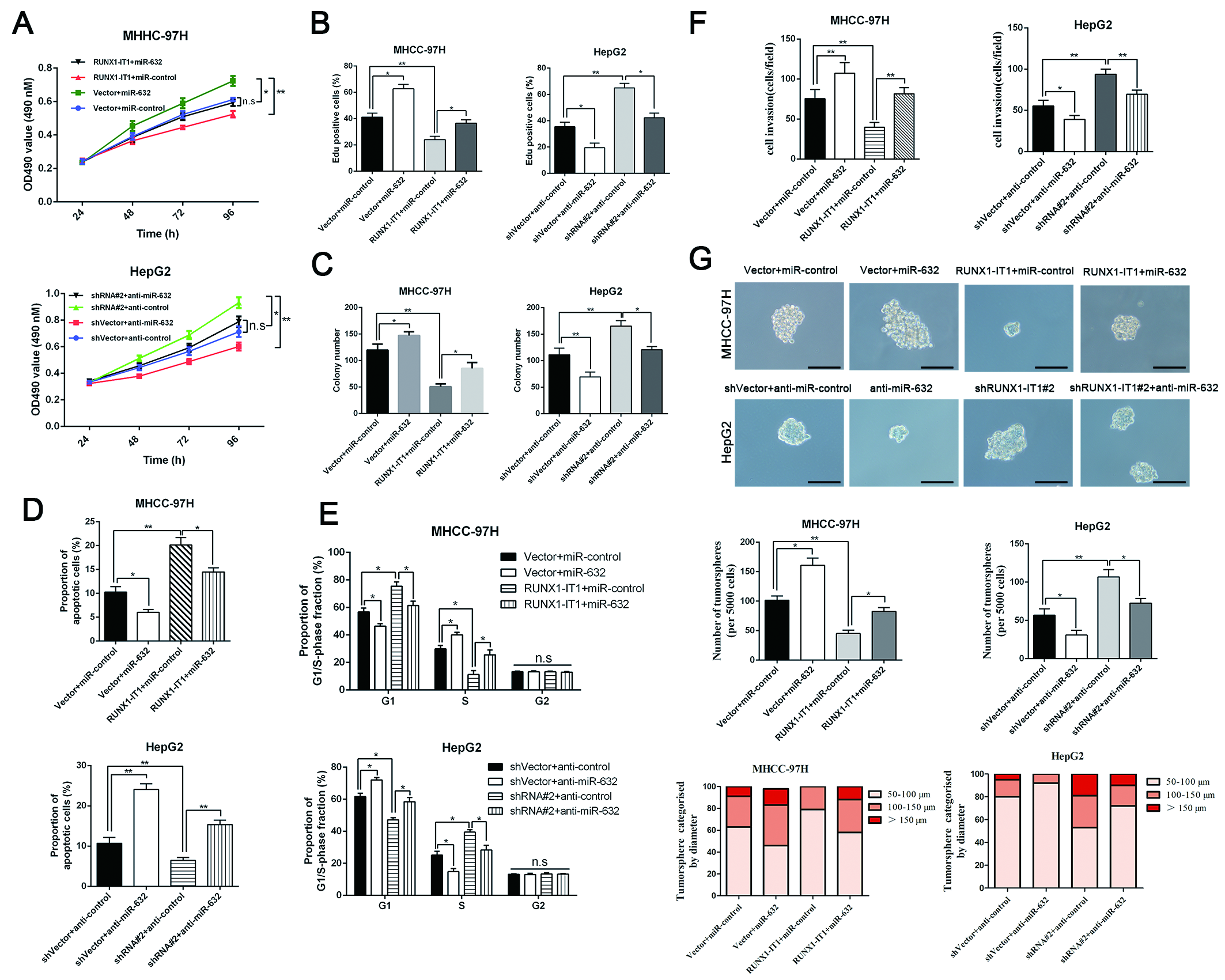

Supplement: Supplementary file 8 — Supplementary Figure 4 [file 41419_2020_2274_MOESM8_ESM.tif]

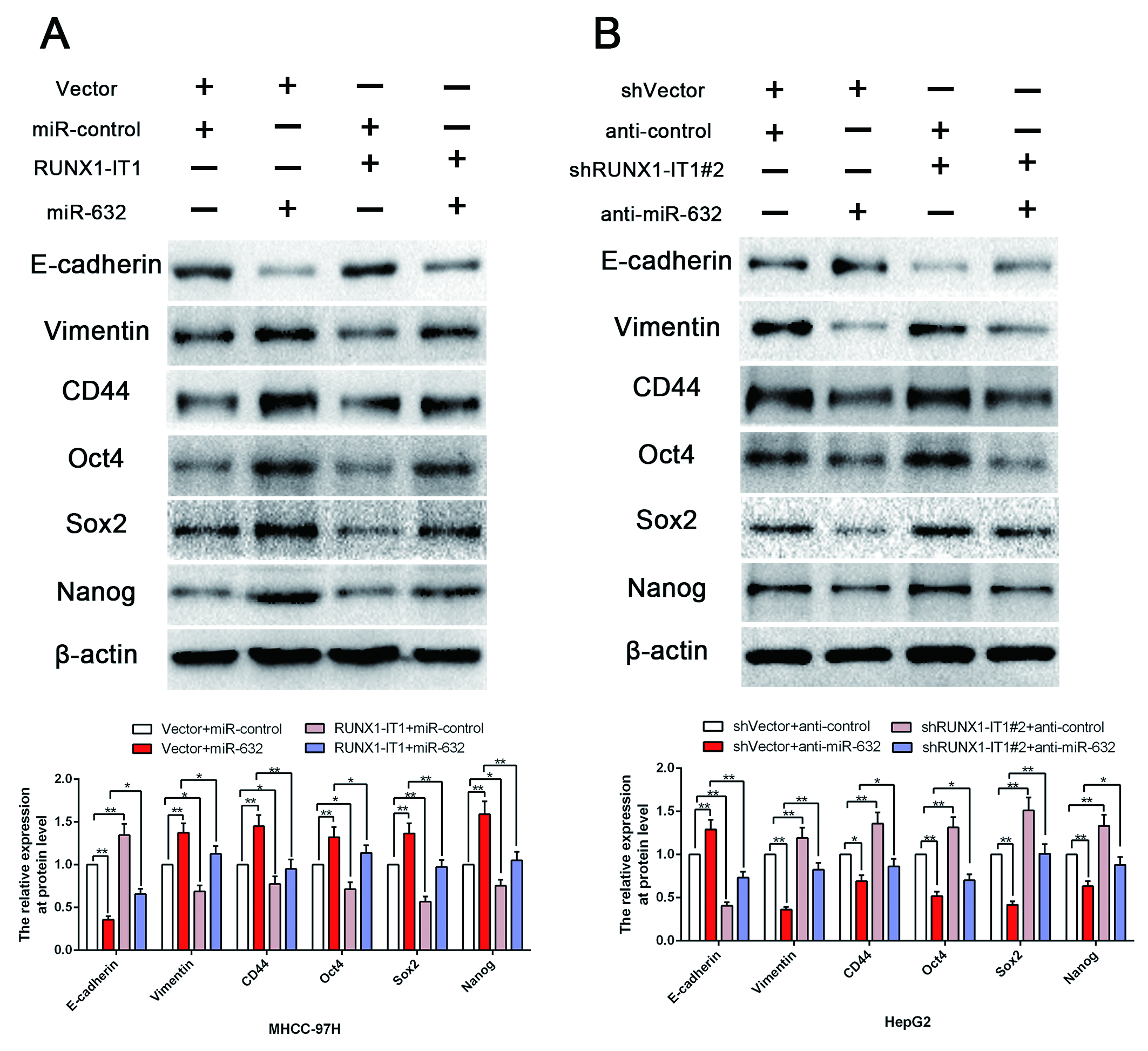

Supplement: Supplementary file 9 — Supplementary Figure 5 [file 41419_2020_2274_MOESM9_ESM.tif]

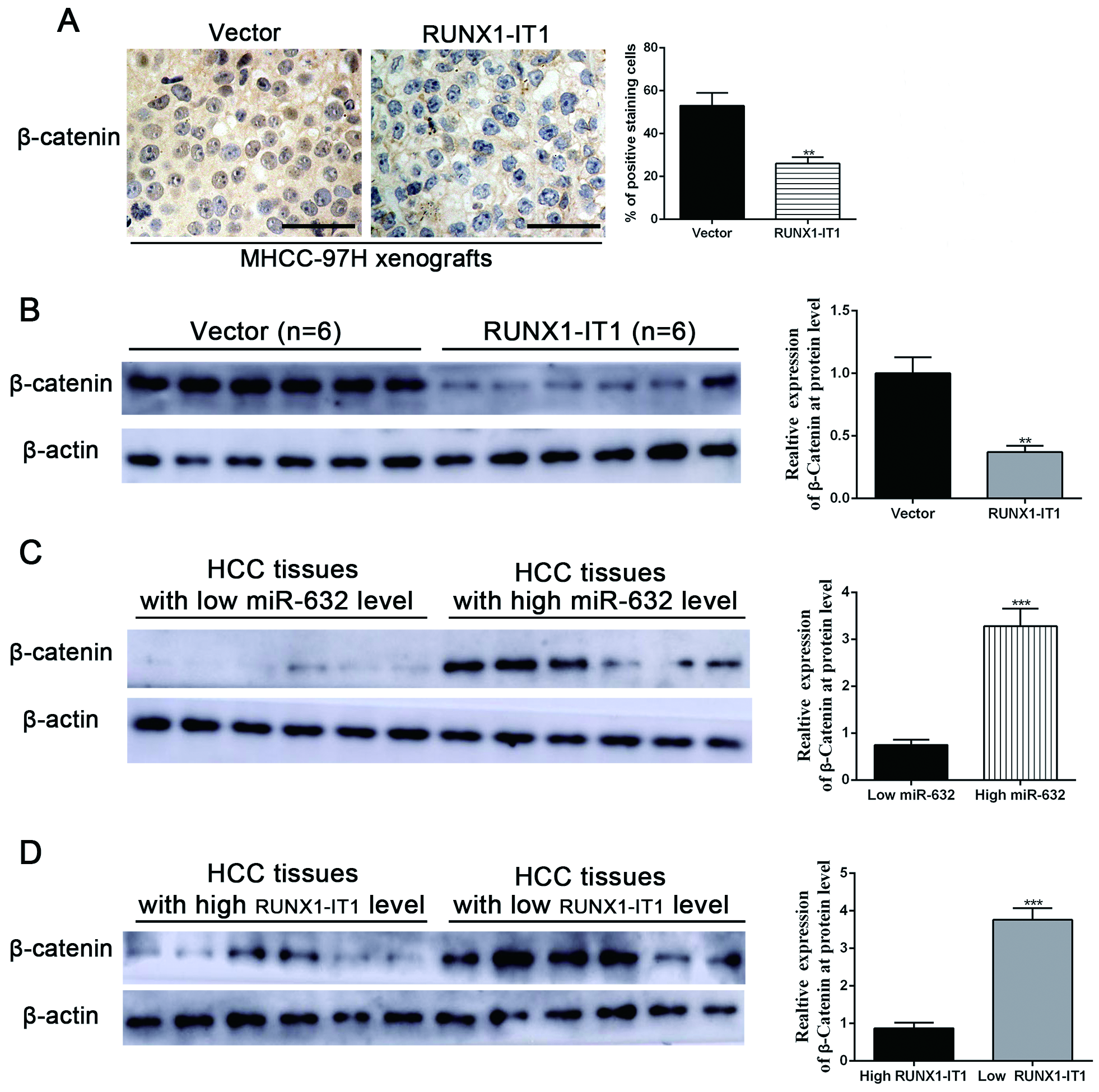

Supplement: Supplementary file 10 — Supplementary Figure 6 [file 41419_2020_2274_MOESM10_ESM.tif]

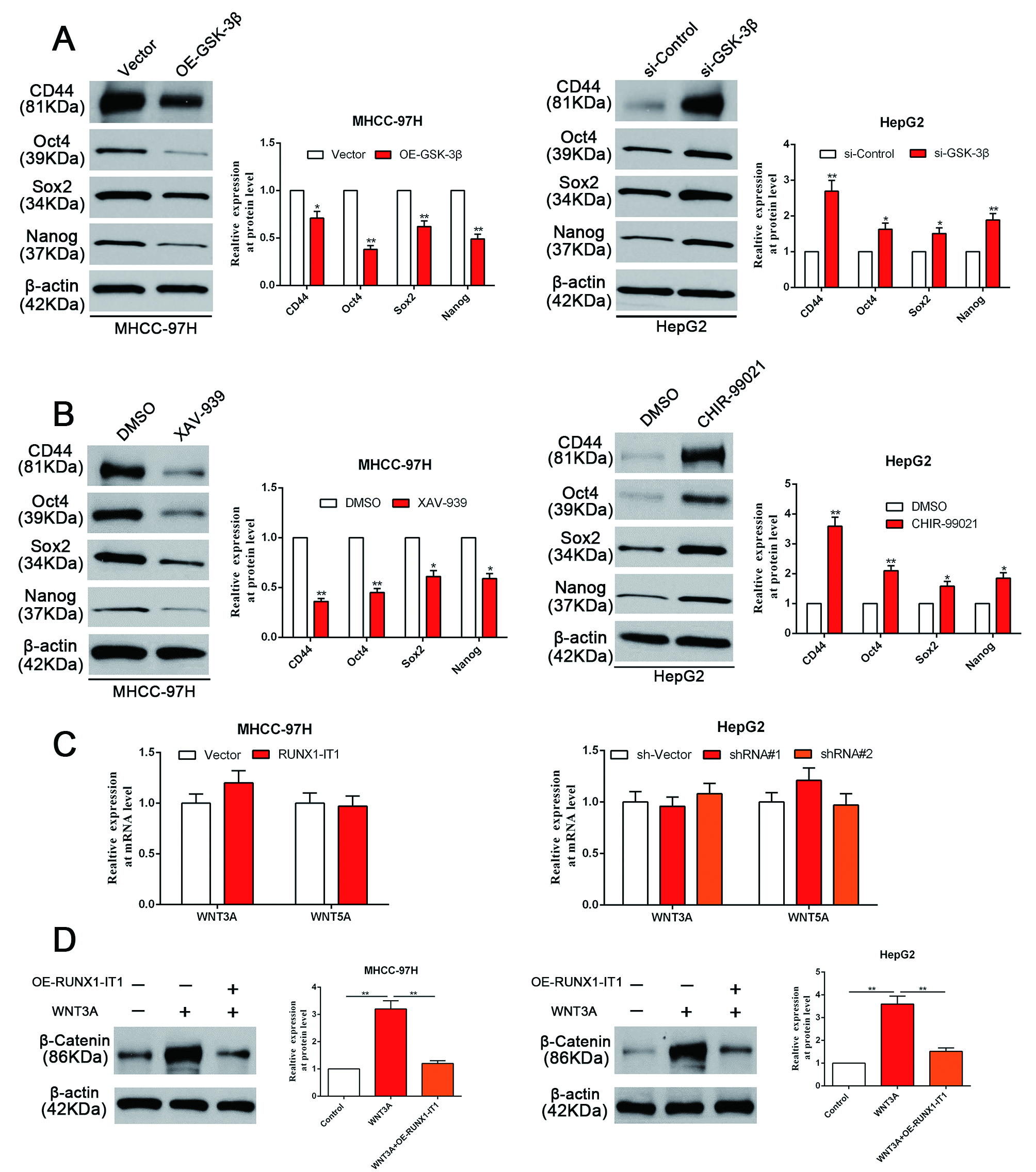

Supplement: Supplementary file 11 — Supplementary Figure 7 [file 41419_2020_2274_MOESM11_ESM.tif]

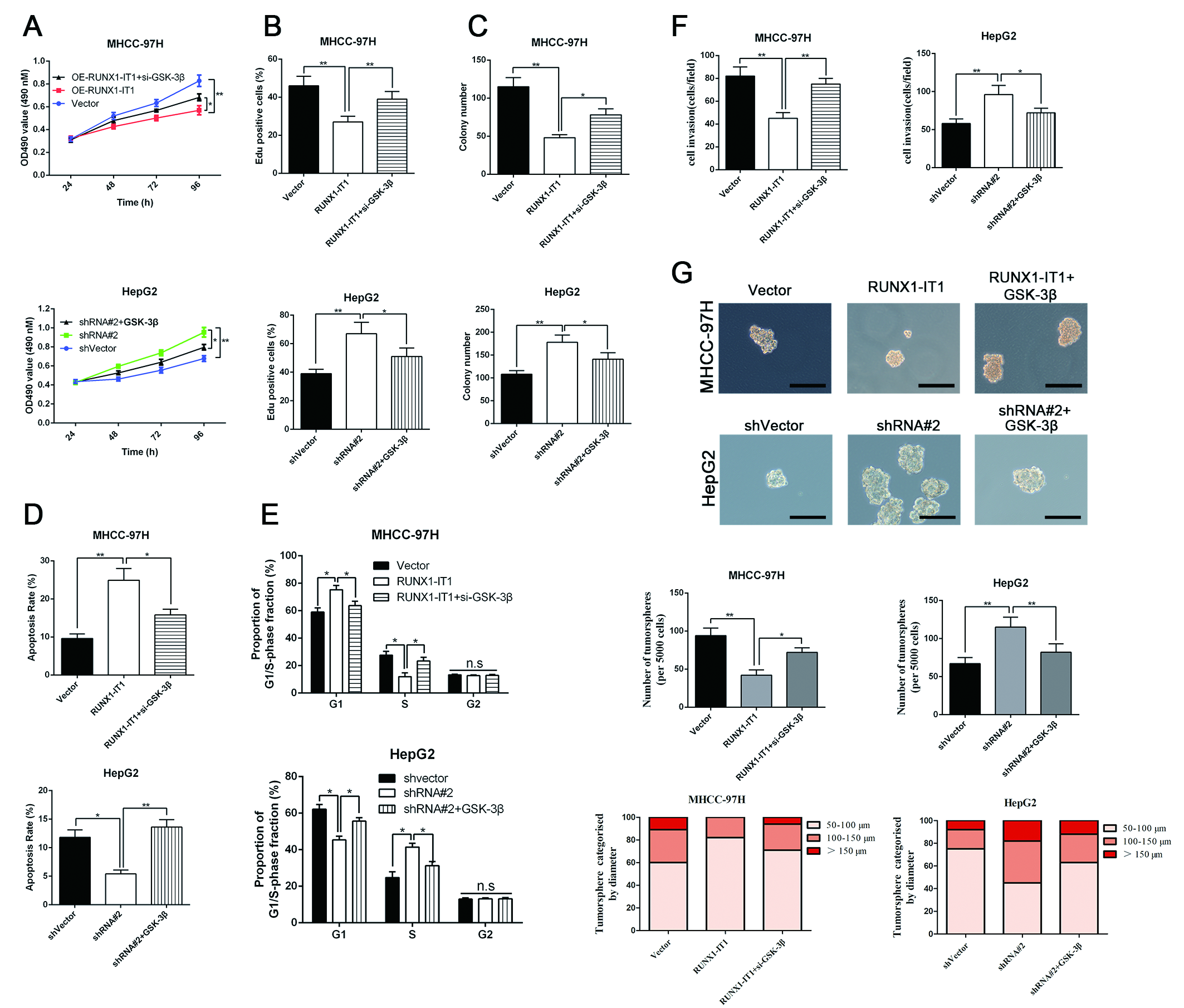

Supplement: Supplementary file 12 — Supplementary Figure 8 [file 41419_2020_2274_MOESM12_ESM.tif]

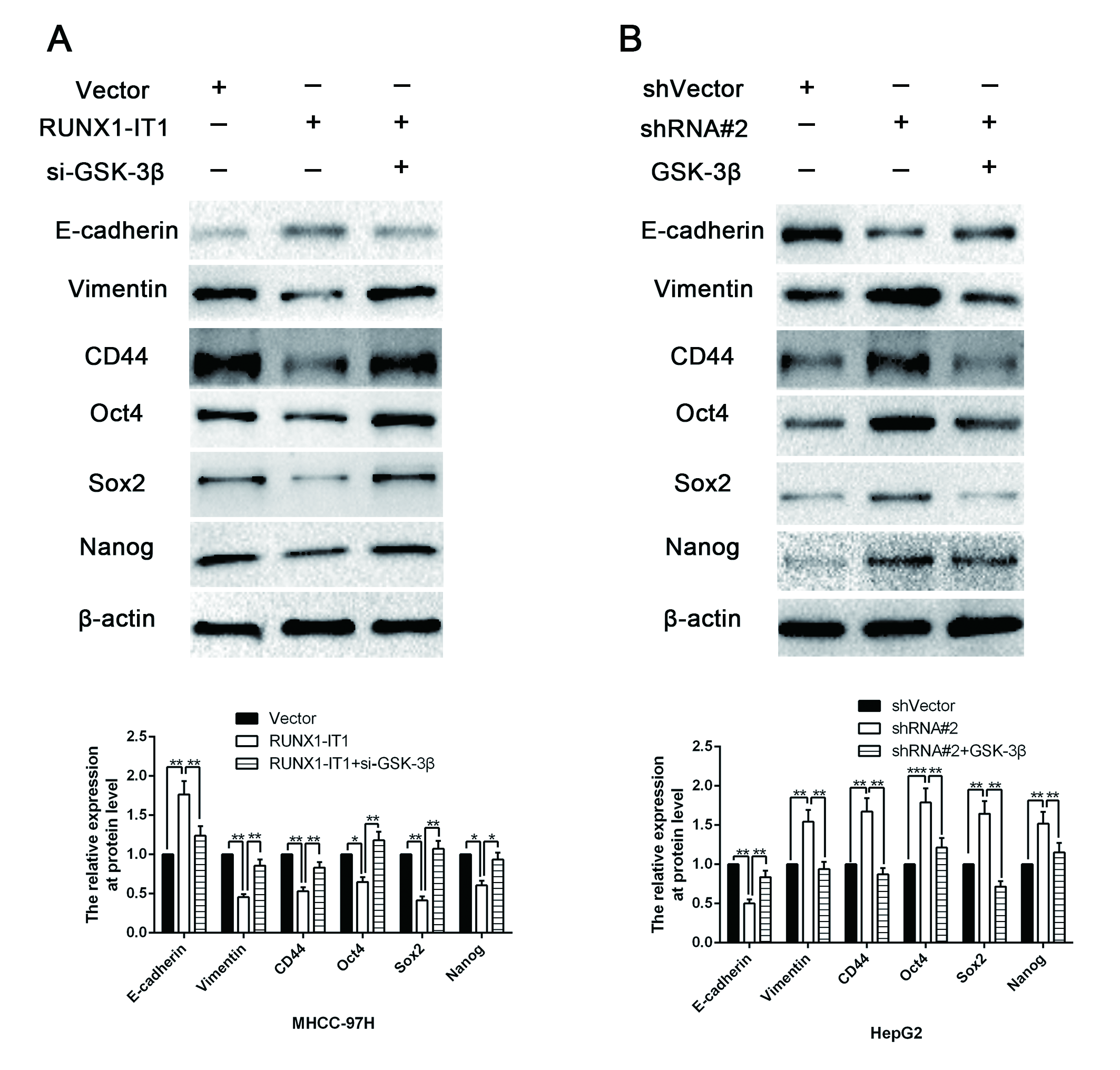

Supplement: Supplementary file 13 — Supplementary Figure 9 [file 41419_2020_2274_MOESM13_ESM.tif]

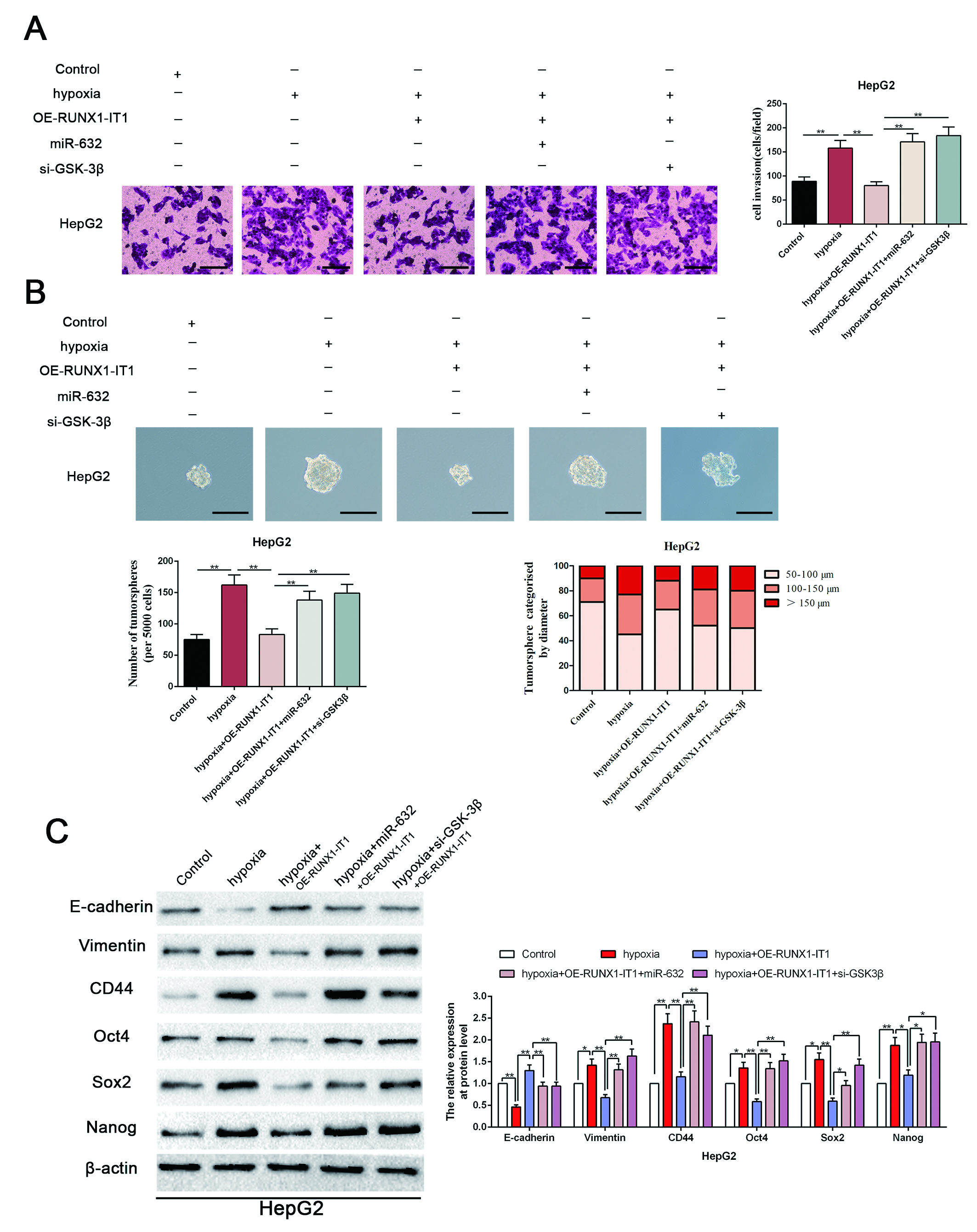

Supplement: Supplementary file 14 — Supplementary Figure 10 [file 41419_2020_2274_MOESM14_ESM.tif]
